# Development and preliminary validation of a patient-report experience measure of acute oncology: The PREMAC study

**DOI:** 10.1371/journal.pone.0359781

**Published:** 2026-10-27

**Authors:** Richard Wagland, Anna Hurley-Wallace, John Defty, M. Jamal Hossain, Alison Richardson

**Affiliations:** 1 School of Health Sciences, Faculty of Environmental and Life Sciences, University of Southampton, Southampton, Hampshire, United Kingdom; 2 University Hospital Southampton NHS Foundation Trust, Southampton, Hampshire, United Kingdom; 3 NIHR Applied Research Collaboration (ARC) Wessex, Southampton Science Park, Innovation Centre, Southampton, United Kingdom; Murcia University, Spain, SPAIN

## Abstract

**Background:**

Patients with cancer often experience oncological emergencies requiring immediate care, and the quality of acute oncology (AO) care varies between providers. Patient-reported experience measures (PREMs) are a key tool for quality improvement, but none currently exists for AO. This study developed a prototype PREM, the Patient-Reported Experience Measure for Acute OnCology (PREMAC), and conducted preliminary single-site psychometric testing.

**Materials and methods:**

A quality assessment framework guided development across three stages. Stage 1 generated a conceptual framework of accessing AO services from a scoping review and interviews with patients and health professionals. Stage 2 developed a prototype questionnaire, with content validity assessed by a Service User Reference Panel (SURP) and face validity tested through cognitive interviews. Stage 3 tested acceptability, feasibility, structural validity and internal consistency at one tertiary cancer centre in southern England.

**Results:**

Data integration identified ten domains, in the chronological order patients accessing AO experience them: reasons for accessing AO; patient preparedness; accessibility of care; the care environment; competence of care; communication and decision-making; compassionate care; the carer’s role; patient anxiety; and service issues including discharge. The 46-item prototype was completed by 171 respondents. Exploratory factor analysis of 18 four-category ordinal items, using polychoric correlations with minimum residual extraction and oblimin rotation (n = 121 complete cases), supported three provisional domains: information, communication, involvement and perceived care quality (α = 0.935); care environment, dignity, trust and safety (α = 0.711); and discharge information and preparedness (α = 0.704).

**Conclusion:**

PREMAC is the first PREM developed specifically for AO. Preliminary testing indicates the prototype is acceptable and feasible, with early evidence of internal coherence across three provisional domains at a single site. The domain structure and item allocations remain provisional; no total score is recommended. Multisite validation is required before PREMAC could support service comparison or benchmarking.

## 1. Introduction

Almost 20 million new cases of cancer were diagnosed globally in 2022, and cancer is expected to become the leading cause of premature death worldwide [1]. With treatment advances, the numbers of patients with a cancer diagnosis receiving treatment within community settings are increasing [2,3]. There is a corresponding increase in patients presenting with symptoms consequent to disease progression (i.e., pulmonary embolism, pleural effusion, malignant spinal cord compression) and treatment-related side effects from systemic anti-cancer therapy (i.e., neutropenic sepsis, acute kidney injury, electrolyte imbalances), or radiotherapy (i.e., diarrhoea, nausea and dehydration, pneumonitis). Such symptoms adversely affect patients’ psychological, physical, functional status and quality of life [4], but they may require immediate specialist cancer care to prevent death [3]. Moreover, patients experiencing such symptoms and their relatives will also often experience high anxiety when they access emergency care, often in stressful, unfamiliar environments [5].

Patients experiencing symptoms indicative of an oncological emergency often seek help at emergency departments [3,4]. However, over the last decade and a half there has been an increasing interest world-wide in developing new approaches to support patients with cancer when they experience symptoms that concern them, to minimise toxicity, improve quality of life, and streamline access to specialist urgent and emergency care [6–9]. Acute oncology (AO) has developed as a new multi-professional approach to unscheduled cancer care, integrating the disciplines of oncology, emergency, acute, and palliative care, to provide cohesive treatment for people presenting to services who are at risk of oncological emergencies [10,11]. However, funding has been described as ‘piecemeal’ and provision varies significantly (13, p8); typically led by cancer clinicians, models of care include telephone and advisory services, AO specialists embedded within other acute care settings (i.e., same-day emergency care), to the provision of standalone AO assessment units [12]. Variations in service provision and quality of care between service providers may affect both patient experience and outcomes [5].

Surveys that incorporate patient-reported experience measures (PREMs) have become an established method for health care providers to measure and understand the quality of service from a user’s perspective [13,14]. Surveys using PREMs enable the experiences and views of representative numbers of patients to be fed back to service providers, information essential for improving services [15]. There are several existing PREMs for use in emergency departments (ED) [16–19], and with cancer patients more generally, such as the National Cancer Patient Experience Survey (NCPES) [20] and the Consumer Assessment of Healthcare Providers and Systems Programme (CAHPS) of the Agency for Healthcare Research and Quality (AHRQ) [21]. However, these do not fully address the specific issues related to patients with cancer experiencing an oncological emergency, which include patient preparedness for help-seeking via AO telephone triage, discharge planning and information provided to patients on symptoms to watch for that should trigger future contact with the AO team [22]. This article reports the development and preliminary single-site psychometric evaluation of a prototype PREM designed for use with patients accessing AO, the Patient-Reported Experience Measure for Acute OnCology (PREMAC). Should subsequent multisite validation prove successful, PREMAC could in future help service providers to identify areas for improvement and to measure change from the patients’ perspective.

## 2. Materials and methods

Frameworks exist to evaluate quality of patient-reported questionnaires and determine usability in a patient population. For this study, the Quality Assessment Criteria framework devised by Pesudovs et al. (2007) [23] was used, specifically the stages up to and including the development of a prototype PREM within one site. The study involved three sequential stages: (1) generation of a conceptual framework; (2) questionnaire development, and (3) a feasibility study. An advisory group was formed comprising patient representatives, AO clinicians, and representatives from Wessex Cancer Alliance and the UK Acute Oncology Society (UKAOS) and consulted throughout the project. A Service User Reference Panel (SURP) was also recruited with assistance from a Patient and Public Involvement & Engagement (PPIE) Officer employed by a service provider, which comprised four people with cancer who had recent experience with accessing AO services.

### 2.1 Ethics

Ethical approval for the study was secured from the National Research Ethics Service (NRES) Committee North West – Liverpool Central, United Kingdom, on 04/05/2023 (23/NW/0120).

### 2.2 Stage 1: Generation of conceptual framework

A conceptual framework of patient experience of accessing AO services during an emergency was developed, informed by findings from a scoping review and interviews with patients and healthcare professionals (HCPs) [23]. The scoping review identified published articles that reported the experience of patients with cancer when accessing urgent care for a suspected oncological emergency. Findings informed both the conceptual framework and topic guides for subsequent interviews. The review’s methods and detailed findings are published elsewhere [22].

Semi-structured interviews were conducted with patients and AO healthcare professionals (HCPs) and managers at a single tertiary cancer centre in southern England, with the purpose of gathering views regarding features of AO care important to patient experience during an episode of care. Eligible patients (>18 years, with cancer diagnosis who had recently accessed AO services) were identified and initially approached by research nurses to determine their interest in participation in the study and provide them with an information sheet. Recruitment took place between 25^th^ September 2023 and 27^th^ November 2023. If eligible patients were interested in being involved, and with their permission, their contact details were passed to a member of the research team (AH-W), an experienced qualitative researcher, who had no previous relationship with participants. AH-W contacted them and arranged a time to conduct an online interview via Microsoft Teams. Immediately prior to the interview taking place, verbal consent was recorded.

Potential HCP participants were recruited between 24^th^ July 2023 and 12^th^ December 2023, identified and initially approached by a member of the research team (JD), a clinical academic with over 10 years of experience delivering AO services. JD contacted potential participants by email; the information included in the email introduced them to the study, outlined what would be expected of participation, and invited them to respond if they wished to take part (and have their contact details passed on to AH-W). A reminder email was sent if no response had been received in two weeks, after which no further reminders were sent. Those who gave permission for their details to be passed on were contacted by AH-W, who followed the same enrolment procedures for patient participants described above.

### 2.3 Stage 2: Questionnaire development

Data from the scoping review, patient and staff interviews were integrated by RW and AH-W to develop a conceptual framework, using the triangulation method (Farmer et al 2006). Once developed, the conceptual framework informed the design of an initial suite of prototype question items. The initial draft question set was structured under subheadings that related to the conceptual framework domains, and discussed with the SURP to ensure each were adequately addressed. The question items were subsequently sequenced to follow the process of a typical patient journey through an AO episode of care, with both ‘routing’ questions that established different stages of the patient journey (i.e., ‘Which service or individual did you first contact?), and evaluative patient-experience questions informed by the conceptual framework (i.e., ‘Were you given enough help on the phone that resolved your problem?’). Response options to the evaluative questions followed a four-point ordered scale (i.e., ‘Yes, definitely,’; ‘Yes, to some extent’; ‘Not really’; ‘No, not at all’). A four-point scale without a neutral option was selected to encourage respondents to indicate a clear positive or negative experience and to reduce respondent burden, which is common practice in patient experience surveys. Members of the SURP tested the content validity of the draft question items, i.e., the extent to which the question items of an instrument are relevant to representatives of the target population [24].

A revised suite of question items was then tested for face validity using cognitive interviews with three patients with cancer, two of whom had participated in the earlier interviews (Table 1). Face validity is the extent to which question items measure what they are intended to measure and can be tested by a sample of the population group for which the questionnaire is intended [23]. To that end, items should be fashioned in simple, unambiguous terms. Cognitive testing was conducted via individual, unscripted online interviews, using Microsoft Teams videoconference, in a think-aloud style [25]. The recall period was defined for respondents on the first page of the questionnaire as ‘your most recent contact with the doctors and nurses who look after you if you have an emergency that is related to your cancer or your cancer treatment’, and the questionnaire was sent one to two weeks after the end of the episode of care.

**Table 1:** Characteristics of participants.

Patient interviews (n = 8)										Cognitive interview
	Primary Diagnosis	Treatment	Reasons for AO attendance	Gender	Age group (Years)	Ethnicity	Disability	Long-term condition	Outcome	
P1	Diffuse large B-cell lymphoma	Chemo-immunotherapy	Constipation Chemotherapy-related fatigue	Male	65-79	White British	Nil	Type 2 diabetes melitus; hypertension	Admitted	No
P2	Metastatic colon cancer	Chemo-immunotherapy	Immunotherapy-related skin toxicity	Male	50-64	White British	Dyslexia	Sarcoidosis Hypertension	Discharged	Yes
P3	Metastatic colon cancer	Neoadjuvant Chemotherapy	Lower limb venous and lower limb thrombus	Female	65-79	White British	Nil	Hypertension Obesity; Osteoarthritis	Discharged	Yes
P4	Advanced stage cutaneous T-cell lymphoma	Chemotherapy	Cellulitis	Male	65-79	White British	Nil	Sezary syndrome; osteoarthritis	Admitted	No
P5	Early stage testicular seminoma	Adjuvant chemotherapy	Upper respiratory tract infection	Male	25-49	White British	Nil	Nil	Admitted	No
P6	Locally advanced sigmoid cancer	Neoadjuvant chemotherapy	Palmer-plantar erythrodysesthesia	Male	50-64	White British	Nil	Hypertension; chronic lower back pain	Discharged	No
P7	Marginal zone lymphoma	None	Lower urinary tract infection	Female	50-64	White British	Nil	Multiple sclerosis	Admitted	No
P8	Metastatic prostate cancer	Hormone therapy	Cancer-related bone pain	Male	65-74	White British	Nil	Psoriasis; hypertension	Discharged	
P9	Chronic lymphocytic leukaemia	Chemo-immunotherapy	Headache	Female	50-64	White British	Nil	Depression	Discharged	Yes
**Staff interviews (n = 8)**										
	**Role**	**Years AO experience**								
S1	Palliative care CNS	10								
S2	Senior Sister (AO)	3								
S3	Specialist medical oncology Registrar (AO)	4								
S4	AO (non-clinical) Service Manager	1								
S5	Senior Lead ACP Lead	9								
S6	Trainee AO ACP	5								
S7	Trainee AO ACP	2								
S8	Senior Staff Nurse AO	2								
**Service User Reference Panel (SURP) (n = 4)**										
	**Primary Diagnosis**	**Treatment**	**Reasons for AO attendance**	**Gender**	**Age group** **(Years)**	**Ethnicity**	**Long-term condition**			
1	Breast cancer	Mastectomy, chemotherapy	Pyrexia	Female	65-74	White	N			
2	Carer of son with Hodkinson’s lymphoma (MM)	Chemotherapy, Immunotherapy, Radiotherapy, Surgery (Transplantation)	Pyrexia Sepsis	Female	60-65	White	N			
3	Lung cancer	Chemotherapy Surgery, Radiotherapy	Neutropenic scepsis	Female	50-64	White	Peripheral neuropathy			
4	Acute myeloid leukaemia	Bone marrow stem cell transplant chemotherapy	Nausea & vomiting	Female	50-65	White	Impaired hearing (secondary to chemo)			

### 2.4 Stage 3: Feasibility study

Validity and acceptability of the prototype PREMAC questionnaire were assessed at one tertiary cancer centre in the south of England over a three-month period (7^th^ May 2024–12^th^ August 2024). Eligibility included all patients ≥18 years old, with a cancer diagnosis who had accessed AO services one to two weeks previously. Those with no confirmed diagnosis of cancer were excluded. Patients were invited to participate if they had sought telephone advice from the AO helpline or attended the AO assessment unit. Eligible patients admitted as in-patients following attendance at the AO assessment unit were invited once discharged.

Eligible participants were identified each week by a data analyst employed by the tertiary cancer centre, using the Somerset Cancer Register (SCR), and duplicates from previous weeks of data collection were omitted. Identification and invitation were undertaken within the care provider organisation, and the research team received only anonymised response data. Potential respondents were sent a text message containing a link to the online PREMAC questionnaire, sent to the mobile number held in their clinical record, from the service provider’s in-house survey platform. The questionnaire was preceded by a participant information sheet (PIS) and a tick box indicating consent, and completion and submission of the questionnaire was taken to imply consent. Responses were anonymous and were not linked to clinical records or to identifiable demographic data. Because responses could not be traced to individuals, reminders could not be sent to non-responders. Patients were invited only after their episode of care had concluded, and the questionnaire asked about the experience of care rather than prognosis or clinical outcome. The PIS provided a link and phone number for the on-site Macmillan Cancer Support Centre, which participants could contact if distressed.

## 3. Analysis

### 3.1 Stage 1: generation of conceptual framework

Interview data were analysed using the Framework approach [26,27], a systematic, flexible approach to qualitative data analysis in health research. Analysis followed seven steps: transcription, data familiarisation, coding, developing an analytical framework, applying the framework (indexing), charting data, and interpretation. A coding framework containing major domains and sub-domains was initially developed using open coding on the first six interview transcripts, split equally between patients and HCPs. A coding framework was then agreed between AHW and RW, and subsequently applied to the remaining 10 transcripts, with flexibility for new codes to be added to each major domain as needed. Once all data had been indexed, the framework was reviewed and condensed into a final set of domains. Themes were then generated by considering relationships and patterns within the framework, alongside researcher notes, PPI insights, and wider research team discussion with the advisory group. The resulting framework of themes was cross-matched with themes identified via the scoping review [22] to identify consistency.

The conceptual framework was developed by triangulating data from the scoping review and participant interviews [28]. The triangulation method involved: coding and comparing findings from each data source; creating a ‘convergence coding matrix,’ whereby data were interrogated for convergence, complementarity, dissonance and silence across sources; developing meta-themes from the matrix that cut across the findings from each data source.

### 3.2 Stage 2: Questionnaire development

The initial question suite was discussed with SURP members at a first content validity meeting in December 2023 to determine their comprehensiveness. Following this meeting, revised question items were re-arranged to form a sequential structure to follow the trajectory of a patient through their AO journey, from ‘First contact’ to ‘Discharge home’ and included instructions for respondents. SURP members were asked to rate these items in terms of their relevance, importance and clarity [29], on a 4-tier Likert scale (1 = ‘Not clear/ relevant/ important; 4 = Highly clear/ relevant/ important) (S1 Table). Each item was then separately discussed at a second online meeting with SURP members in January 2024. Items were to be retained if the mean score between members was ‘3’ or ‘4’ for all criteria (clarity/ relevance/ importance) and would be retained but revised if they scored a mean of ‘3’ or ‘4’ for relevance and importance but lower for clarity. Items could be removed if, following discussion, the consensus rated them lower than ‘3’ for either relevance or importance. A further discussion would have taken place between the Advisory Group before items were removed, but in the event this was unnecessary. SURP members could suggest the addition of items [30], and if there was a consensus following discussion that rated them ‘3’ or ‘4’ for importance and relevance, then they were added to the prototype questionnaire.

The questionnaire was then cognitively tested by three patients at separate online meetings conducted in February 2024 using the Read Aloud approach. Cognitive interviews were facilitated by AH-W, who wrote discussion summaries. A final content validity discussion was then conducted via email with SURP members in March 2024, and comments and suggested revisions from cognitive interviewees were reviewed.

### 3.3 Stage 3: Feasibility study

Descriptive statistics were used to summarise participant characteristics and item-response distributions. The 18 evaluative items selected for exploratory subscale analysis (Q13–Q25 and Q32–Q36) used four ordered response categories, coded from 1 (‘No, not at all’) to 4 (‘Yes, definitely’), with higher scores indicating a more positive experience. PREMAC has a mixed measurement structure; routing, categorical, carer-specific, global, free-text, clinical and sociodemographic items were therefore not included in the exploratory factor analysis (EFA). Full item classifications and reasons for inclusion or exclusion are provided in Table 4.

Because most items demonstrated ceiling effects and negative skewness, the primary EFA used a polychoric correlation matrix, minimum residual extraction and oblimin rotation, allowing factors to correlate. The polychoric matrix required smoothing before extraction. Sampling adequacy was assessed using the Kaiser–Meyer–Olkin measure and Bartlett’s test of sphericity calculated from the Pearson correlation matrix. Factor retention was informed by parallel analysis using 500 iterations and the 95th-percentile criterion, inspection of the scree plot and substantive interpretability. A standardised pattern coefficient of at least 0.40 was considered a salient primary loading. Secondary loadings of at least 0.30 were reported, and items were classified as cross-loading when two or more coefficients were at least 0.30 and differed by less than 0.20.

The primary analysis used complete scorable responses across all 18 items (n=121); no imputation was performed. Non-scaled responses to Q19 and Q22 were treated as non-scorable. Sensitivity analyses examined available-case estimation, alternative coding of non-scaled responses, Pearson correlations with maximum-likelihood extraction and Promax rotation, and inclusion of Q11 and Q12; full details are provided in S2 and S3 Tables.

Internal consistency of the provisional domains was assessed using Cronbach’s alpha. Ordinal alpha, McDonald’s omega and item-level reliability statistics are reported in S4 Table. Alpha was interpreted as a reliability coefficient rather than evidence of structural validity. Reporting was informed by relevant COSMIN principles, recognising that PREMAC is a patient-reported experience measure. All analyses were conducted in R version 4.4.2 using the psych package (version 2.4.6.26) for polychoric correlations, factor analysis and reliability estimation, and GPArotation (version 2024.3−1) for oblique rotation. The annotated analysis script is provided as S1 Script.

## 4. Results

### 4.1 Stage 1: Generation of conceptual framework

#### 4.1.1 Scoping review.

Sixteen articles reporting on 14 unique studies were included. Issues important to patient experiences of emergency cancer care were described by patients, healthcare professionals and carer proxies [22]. Studies captured experiences of various care delivery models including telehealth, in-person presentation to an Emergency Department and AO services. Seven core domains of patient experience arose from the synthesis: managing emotional distress, palliative care discussions, being treated with compassion and respect, deciding when to seek help, hospital environment, quality of care and communication, and discharge planning.

#### 4.1.2 Interviews.

A total of 16 interviews took place. Patient participants (n = 8) included six men and two women, with a range of ages, types (and stages) of cancer, treatment modalities, and acute symptoms and conditions that required seeking help from AO services (Table 1). HCP participants (n = 8) included a range of clinicians (i.e., a medical oncology registrar, palliative care clinical nurse specialist (CNS), acute oncology advanced care practitioners and service manager), with a range of experience working within AO from 1 to 10 years (mean: 4.5 years). Eighteen themes were generated from the interview data. Table 2 identifies the list of themes, and illustrative extracts from interviews. All themes were coded for both patient and HCP participants.

**Table 2:** Conceptual framework dimensions, qualitative interview themes, and illustrative quotes.

Conceptual framework domains	Definition	Interview themes	Examples from qualitative interviews
**Reasons for accessing AO**	The symptoms and problems related to cancer and treatment with which patients present in AO.	Reasons for accessing AO	Patients sometimes described the symptoms they had experienced, and which prompted them to seek help from AO services: ‘*You have no idea what it’s like at five o’clock in the morning not being able to go to the loo, in either sense, and it’s just incredibly uncomfortable*.’ (Patient) Staff outlined the range of symptoms and problems that patients presented with when they contacted the telephone helpline or were referred to AO services: ‘*They come in for… lots of, many different medical complications or issues for their treatments. Some are, for example, query spinal cord compression and query neutropenic sepsis and pain management*.’ (HCP) ‘*From a palliative care perspective, it’s usually about pain and symptom management. So pain, nausea, constipation*.’ (HCP) Staff also noted that, given the development of new treatments, increasing numbers of patients will experience treatment side effects and require AO services: ‘*what we’re looking into, is that in the next year, 52 more immunotherapies are coming out, which is gonna cause a lot more side effects*.’ (HCP)
**Patient preparedness**	Patients’ knowledge of the AO service before a problem arises, whether they know who to contact, when, and for what problems	Patient preparedness (knowing who, when and what to seek help for)	Some patients were unsure who to contact (‘*I think first I may have contacted the lymphoma nurses and they redirected me to Macmillan*,’ (Patient)) Most patients had been provided with an emergency number, and were given some guidance about what symptoms to call about: ‘*it was on the paperwork that if the temperature went above 38 I ring this number. So we rang the number and we were sort of told what to do*.’ (Patient) Patients sometimes struggled to know when to seek help for a problem or for which problems they needed to seek help, partly because the advice they had received was vague or because they had not been able to process and understand the advice: ‘*I’ve got it [Information pack] in front of me actually. But I don’t think there was more context given, I think it was just a number you should ring if you have a real big problem. …. But when you first got the diagnosis you’re knocked for six and you’re bewildered by just about everything so if you take it in it’s a goddamned miracle*.’ (Patient) Staff noted patients were often unsure what problems to seek help for, and help-seeking delays often led to problems becoming severe: ‘*even if they’re having palliative chemo, they are told that if they’ve got a temperature then to call about that. What they might not think about calling until it’s sort of further on down the line is if they haven’t opened their bowls for medication, that’s likely to constipate them and then they end up being having more pain because they’re constipated. So the ‘what’ is quite hard to sort of identify and quantify*.’ (HCP) Staff commented patients often found it difficult to navigate the care system and from whom to seek help: ‘*being at home they can feel quite isolated, even though they’ve got very specialist nurses to call, you know, especially if they’re early in their journey, they haven’t found out how to navigate their way around. especially early on in their disease trajectory, you know, everything’s new, they don’t really know what’s normal anymore for them*.’ (HCP)
**Accessibility of care**	Accessibility and timeliness of assessment and treatment.	Assessment & admission	Most patients had sought help firstly through the AO triage Help-line. Staff commented they aim to assess and triage patients promptly over the phone to determine whether they should attend the AO unit, and patients reported that prompt assessment is what they experienced: ‘*I just followed the instructions, as I say, where I phone in and they triage me over the phone*.’ (Patient); ‘*patients can phone or relatives can phone the acute oncology service and then they get triaged and then either given advice or told to come in*.’ (HCP) Triage and assessment of attending patients then occurred within the unit. ‘*prompt admission and sort of prompt service when they arrive, so prompt OBS, bloods, assessment. And actions, whatever needs to be taken. So if they need to go for scans, making sure that’s done in a timely fashion.*’ (HCP) ‘*we do triage on acuity. So we would understandably, like any assessment area, go to and care for the patients that are triaging red rather than green first*.’ (HCP) Patients in the sample reported how efficient their assessment had been: ‘*I had to go in twice for a high temperature and once I had poison coming out of my big toe on the nail bed and they were on top of that very quickly and they got that sorted. So, I just can’t fault the treatment, it was very fast and very good*.’ (Patient)
		Equitable care	Staff reported that care was provided equitably and prioritised depending upon need: ‘We want equitable service and experience for everyone, and I think that’s really, really fundamental, for us.’ (HCP4) ‘I’m really passionate about equitability and accessibility. And above all, I think that’s what we should be aiming for. So, no matter who somebody calls at whatever time of day or night, they get access to the same level of care that they would to their cousin that lives down the road.’ (HCP5) Work ongoing to ensure accessibility for patients with various disabilities. ’I’m supporting with the communications team, in terms of a dial-in option to … So, if you have a speech impediment or you cannot talk for any reason, there is an option on that phone to dial and it will relay messages to you. … the front desk isn’t [currently] disability friendly’
**Care Environment**	Layout of AO unit, waiting, privacy, comfort and safety.	Care Environment	Participants commented on several aspects regarding the environment in which AO care was delivered. Limited privacy was identified by both patients and staff, especially as patients might be very unwell, emotionally distressed, using commodes or attending to bodily functions, or engaged in difficult conversations about prognosis and treatment with clinicians often had only: ‘*the privacy of the magic curtains that are totally not soundproof!*’ (HCP) ‘… *sometimes patients who come into AOS are acutely unwell. And sometimes very sick, and you’re having difficult conversations with them with the curtains around and of course that doesn’t give you any privacy*.’ (HCP) ‘*There’s no way that they can have a private conversation unless the side rooms are free. You can’t have a private conversation on AOS at all*.’ (HCP) Patients were very appreciative when they had use of a side room. ‘*Well because of the problem I had, my acute constipation, and the remedies that may produce results at any given time, I had a side room so I was on my own. It’s good in those situations to have your own loo, believe me*.’ (Patient) Staff often commented on the ‘*challenging*’ pressures on beds that existed especially as ‘*a lot of patients are waiting for a bed downstream*’ (HCP). Staff described how the unit had become busier overtime as the number of attending patients had increased: ‘*when I first started three years ago, we had 6 inpatient spaces plus one side room, when we could. Within three years we’ve gone to 16, so we’ve added 10 assessment spaces, so it’s not like the service was built for that – we have gathered [extra spaces] because of the demand of the service*.’ (HCP) ‘*It’s just the service is growing so quickly that we’ve had to gather the environment along the way, which may have not suited the service itself*.’ (HCP) The AO unit could ‘*be quite a chaotic environment at times and lots of noise and lots of chatter*.’ (HCP), which patients can find ‘*intimidating*’. It also means the environment can be uncomfortably hot: ‘*The treatment room is extremely hot, goes above 31 degrees. The door is constantly open*.’ (HCP) A very busy environment can increase waiting times for patients, which negatively impacted upon their experience. ‘*They try and do their best with the situation they’ve got but sometimes… I don’t know how to put this… you wait too long.*’ (Patient)
**Competence of care**	How well the multidisciplinary team works together and competence of clinicians	Multidisciplinary care	Staff commented that the AO team was ‘*a very large multidisciplinary team*’ which meant they can ‘*pull upon the specialties that are needed*.’ (HCP). There were some challenges, such as that ‘*physios, dieticians, SALT [speech and language therapy] referrals and occupational health are more day hours; 8 til 4*.’ (HCP), but the multi-disciplinary nature of the skill mix meant a holistic approach to care could be provided: *‘… a patient comes in, they don’t normally just come in for one thing. … There’s multiple things. The dietitian, they’re not eating properly because of the treatment, so they’re malnourished. They haven’t been getting out of bed because they’ve been really tired. So then their physio, and their bones and muscles are not moving and weaker*.’ (HCP) Patients commented on the level of competence displayed by clinicians within the AO service, and how important this was for their confidence: ‘*You felt that they were experienced and they knew what they were doing. … it matters and, you know, how the nurse behaves matters, and they’re just faultless*.’ (Patient)
**Communication & decision-making**	Communication between clinicians, patients and carers, and the level of information they receive, and the extent to which patients are involved in shared decision-making.	Information preferences	The amount of information patients preferred to receive about their condition, investigations and potential outcomes differed widely. Most patients reported they had received an appropriate level of information: ‘*I was informed, I understood, and I was very happy*’ (Patient). Some patients wanted very little information: ‘*They wanted to give me an explanation but like I told them I’m the kind of person that overthinks things so I asked them to give me as little information as possible*.’ (Patient). Others wanted more specific and in-depth explanations: ‘*Oh yes, I understood, yes. Which is why I had questions. Which is why I felt they should have been answered. … I knew what was going on, I knew what I thought to expect and I needed current information to relate to, to calm my fears a little or just give me possible alternatives other than a bleak outcome*’ (Patient)
		Shared decision-making	The extent to which patients wanted to be involved with decisions about treatment varied between those who wanted control of decisions; *‘Basically speaking I was in control as much as I wanted to be*’ (Patient), to those who preferred to delegate decision-making to ‘*professionals*’. The extent to which patients wished to share decision-making often depended upon how well they felt: ‘*it depends on how sick you are and what you want. Because some people just get there and they let [clinicians] do what they want because they want to just get out of there*.’ (Patient) Staff reported they would encourage patients to ask questions: ‘*I think it’s being encouraging them to sort of ask questions*.’ (HCP)), but they gauged the level of involvement in decision-making a patient would likely prefer by their health status: ‘*So some of the care we give to acutely unwell patients, with consent, we just kind of crack on telling them what we’re doing at the time*.’ (HCP)
		Clear communication	Patients often find it difficult to determine the roles different clinicians have within the AO service ‘*I didn’t know who was in charge of what, I mean, you don’t, you see so many people*.’ (Patient). So, it’s important for clinicians to clearly introduce themselves: ‘*Yes, they introduced themselves. … if anybody had a specific role to perform they told me what they were there for*.’ (Patient) Both staff and patients reported the importance of patients receiving clear explanations of their symptoms and the investigations and treatment they were to receive. Sometimes it takes time for tests to be completed before answers can be given: ‘*They’re coming in because something is maybe not going to plan. So they’re kind of desperate to know the answers there and then, which I think can be challenging at times because you have to do a lot of more investigation to find out what’s going on*.’ (HCP) Sometimes patients felt they were not getting the answers they needed in a timely way: ‘*But they didn’t tell me anything, they just let me sit in there and that’s… I had it out with the registrar that… No feedback, you know, they left me there without talking to me, they just said, “Somebody’ll come and see you” and it was like five hours later*.’ (Patient) Staff sometimes had to have difficult conversations with patients, which is difficult in a busy assessment unit: ‘*And we are the ones that potentially break or approach those conversations, which can be tricky and challenging in assessment area*’ (HCP) Staff reported they would consider the correct way to ‘pitch’ explanations in a way the individual would understand them: ‘*If you’ve got, you know, a university professor compared to a car mechanic their information requirements might be different’* (HCP)
**Compassionate care**	Being treated ‘as a person’ with kindness, staff building a rapport with patients, providing emotional support.	Compassion and understanding	Staff were aware of the anxiety that patients feel when they arrive on the unit and that ‘*patients will want to just feel safe because they trust us to do the best to care for them*’ (HCP), and they need to ‘*feel like they have been understood, listened to, and safely cared for*’ (HCP). One patient stated that ‘*it’s terribly important that the person is recognised as a person, not just a condition*’ (Patient), and several others reported how a caring and compassionate attitude from clinicians, and even a sense of humour, greatly impacted upon their experience: ‘*you’re talked to, they have endless time and patience for you – in that respect it’s wonderful. Because when you’re really scared and you’ve nobody you can talk to and you don’t want to burden your family then having one person sort of come up with the kindness that I received there it does you almost more good than the treatment of antibiotics does. It’s what you naturally need at that time*.’ (Patient) ‘*Not only are they kind but a couple of them have got sense of humour and it was really nice. You know, you can relate to somebody who has a sense of humour, you can’t to a deadpan face, and none of them were like that, they’re all really engaged and really empathetic and really kind*.’ (Patient)
		Advocating for patients	Staff recognised the importance of listening to the patient, reading their non-verbal communication signs to understand their needs and to address these as an advocate: ‘*they may feel that when they come in, potentially they haven’t got any empowerment to their own care or their own bodies. To actually listen and take on board how they feel and what they would like and to be an advocate for them at that time, from the nursing team*.’ (HCP) Patients who had felt vulnerable during their oncological emergency greatly appreciated staff listening and understanding their needs: ‘*Because when you’re really scared and you’ve nobody you can talk to and you don’t want to burden your family then having one person sort of come up with the kindness that I received there it does you almost more good than the treatment of antibiotics does*.’ (Patient)
**Carer’s role**	Carers as support and advocates for patients, and the extent to which clinicians address the needs of carers.	Carer’s role	Several patients reported they had been accompanied by friends, family or partners during their attendance of the AO unit, or that they had taken a lead on help-seeking, and contacting the AO team: ‘*My husband made the phone call after I peaked on my temperature and he just got the information of where to take me and, to be honest, I didn’t know anything really*.’ (Patient) Informal carers provide emotional and practical support for patients and act as their advocates during an oncological emergency. Staff reported being aware that a patient’s carers are often more critical of the care provided than patients and will often be watchful: ‘*So I suspect carers might be a lot more...critical… of the sort of privacy and services and that sort of thing, because they’re sitting there and observing*’ (HCP) Staff recognised carers sometimes found accompanying their loved one while they were ill stressful: ‘*it’s harder being on the outside looking in, for carers, I suspect… and that’s their psychological overlay of their distress as well*.’ (HCP) Staff commented that having an accompanying carer could also be burdensome for patients, if they do not wish to show how unwell or frightened they feel: ‘*actually, sometimes people can come in with their family and their family, understandably, are very anxious and actually make it more challenging for the patient at that point when they’re in coming in with some sort of complication or issue. Actually, I think at times it can be because they’re trying to be strong for their relative, carer, it can be more of a burden on the patient*.’ (HCP)
**Patient anxiety**	Causes of anxiety amongst patients and the extent to which anxiety is addressed by clinicians.	Patient anxiety	All patients described their anxiety during their oncological emergency, which can have several sources. Primarily, patients are unsure what symptoms signify, whether their condition is worsening, how long investigations will take to identify problems and what treatment is necessary: ‘*I think it’s really unnerving for anyone not knowing potentially what it could be. And you know, say, tissues may need to be sent and sent to a lab or blood cultures take more than 24 hours to grow times, for example. So it can be frustrating and understandably worrying for these patients*.’ (HCP) Patients often experience anxiety at the possibility of having to be admitted as an in-patient: ‘*they don’t like the thought of staying, so if they have to be admitted that to them is anxiety-provoking potentially*.’ (HCP). Patients undergoing some treatments, such as chemotherapy, are often worried their symptoms might require their treatment to be stopped, which could delay help-seeking: ‘*the anxiety potentially is something to do with their treatments, either whether they’re worried that it’s gonna be stopped, postponed and if the cancer has progressed*.’ (HCP) Patients need to feel able to express their anxieties to staff and for staff to listen and reassure them. ‘*And patients, when they’re terrified and can’t sort of communicate, they need someone they feel they can talk to, it’s terribly important, because stress is as dangerous as the disease*.’ (Patient) Patients who had a good experience of the acute oncology unit if they had previously attended, sometimes indicated their anxieties diminished on subsequent visits. ‘*I knew when I got in there (acute oncology) after the first time that they’d look after me so I didn’t have principal concerns*.’ (Patient)’ ‘*I don’t like hospitals, I’ve had enough of them in the respect of, you know, having to be there, but I’m not scared to go there (AO unit) and I’m not distressed to go there, I think, “Oh good, they’re going to sort this*.’ (Patient)
**Service issues**	Whether the discharge process is timely and smooth, and how other services both internal and external to UHS are linked with AO, i.e. routes of access in and referrals, transfers and discharges	Care pathways	Some staff participants described several routes patients might take to the AO service. Patients might refer themselves by contacting the cancer helpline: ‘*it might not be that we do a face to face conversation, but we would definitely give some phone advice, so at least they’ve got a starting point*.’ (HCP) Alternatively, patients might be described as ‘internal referrals’: ‘… *which the nurse in charge will coordinate. And these are referrals that are done by anyone that is in the trust. For example, someone on a day unit that’s reacting to a treatment. Someone that’s having radiotherapy that is unable to eat and drink properly and have the really bad nausea, someone that has gone for a CT and picked up something on the result. A regular just pick something on their result, for example, blood clot metastases. So, there’s three different referral pathways into AOS*.’ (HCP) Patients might also be referred ‘*externally*’: ‘*So these are referrals that come from GP’s and doctors, some other hospitals. … And that will go through to a registrar or a senior decision maker who will accept that referral*.’ (HCP)
		Discharge from AO	Staff commented on the need to facilitate discharge of patients as smoothly as possible, for the benefit of patients, but also for the efficiency of the service: ‘*most importantly, is about making sure that the discharge, if they are going home, that’s all done smoothly as well. So, their paperwork and their medication, TTOs, etc. are done in a timely fashion*.’ (HCP)
		Service linkage	Some staff participants discussed the links between AO and other services, and communication across services, particularly palliative care and supportive care in the community, to help patients continue to cope at home: ‘*sometimes it can be that they haven’t got the support at home that they need and that’s what we can help try and link up with the Community palliative care services*.’ (HCP) Staff also highlighted the need for sign-posting patients to other supportive services, particularly with regards their finances: ‘*you know, even if it’s the case of finances, we signpost them to McMillan CAB. You know all those sorts of different [services].*’ (HCP)
Not included in framework		Gratitude to staff	Almost all patient participants were keen to convey their gratitude to the AO service and staff, whose ‘*fantastic*’, ‘*brilliant*’, ‘*caring*’ attitude and approach, despite workloads, who ‘*make it as pleasant an experience as you can expect in those circumstances*’ (Patient).
Not included in framework		The cancer journey	Several patients described their wider cancer journey, their experience of diagnosis and treatment and previous experiences of care within other clinical environments other than AO.

The scoping review and interviews identified broadly similar themes. Ten domains emerged from the triangulation analysis to form the conceptual framework incorporating findings from the review and interviews (Table 3). These domains followed the chronological order in which patients accessing AO would be expected to experience them: reasons for accessing AO; patient preparedness; accessibility of care; the care environment; competence of care; communication and decision-making; compassionate care; the carer’s role; patient anxiety; and service issues (including discharge). These comprised the content area to underpin development of the prototype PREMAC questionnaire.

**Table 3:** Conceptual Framework: Integrated data across the development phases of PREMAC.

Themes generated from codes across datasets	Codes and themes generated from interviews dataset (Patients & HCPs)	Domains identified by scoping review
Reasons for accessing AO	Reasons for accessing AO	Deciding when to seek help
Patient preparedness	Patient preparedness (knowing who, when and what to seek help for)	
Accessibility of Care	Assessment & admission	N/A
	Equitable care	N/A
Care Environment	Care environment	Hospital environment
Competence of care	Multidisciplinary care	Quality of care and communication
Communication & decision-making	Information preferences	
	Shared decision-making	
	Clear communication	
Compassionate care	Compassion & understanding	Being treated with compassion and respect
	Advocating for patients	
Carer’s role	Carer’s role	N/A
Patient anxiety	Patient anxiety	Managing emotional distress
Service issues	Care pathways	Discharge planning
	Discharge from AO	
	Service linkage	
N/A	Gratitude to staff	N/A
N/A	The cancer journey	N/A
N/A	N/A	Palliative care discussions

### 4.2 Stage 2. Questionnaire development

SURP members were all white and female, three had cancer diagnoses and had undergone a range of treatments, and one was the carer for a son with Hodgkins Lymphoma in his twenties. Each had recently attended AO (as patient or carer) (Table 1). While there was variation in ratings given by SURP members to some question items, all evaluative quality of experience items received a mean score of between 3–4 for clarity, relevance and importance. SURP members made several suggestions for additional items. For example, in the section on ‘Discharge’, one SURP member suggested inclusion of an additional question to ask whether the information patients received on discharge was written down (Q36). Another SURP member suggested adding an item asking patients who had delayed seeking help for a symptom why they had done so (Q2). These suggestions were subsequently included.

Not all suggestions were incorporated into the final version. A SURP member suggested combining Q23 ‘*treated with respect*’ with Q21 ‘*feeling listened to by staff*’. Following discussions with other SURP members, research team and advisory group, it was decided these two questions addressed different elements of the conceptual framework domains and should remain separate.

Participants involved in the cognitive interviews also questioned some items and proposed revisions. For example, a linguistic change was suggested by two interviewees to remove the phrase ‘in timely way’ as not understood in non-native English, and replaced with (Q32) ‘Were you happy with the time take for you to be discharged home?’ Finally, additions and changes were suggested for some response options, for example, for the item (Q19) ‘Did the nurses and doctors involve you in decisions about your care and treatment as much as you wanted to be?’, it was suggested to add the option: ‘I did not want to be involved and was happy to take advice from doctors and nurses’. This was added to Q19 of the final version of the prototype.

Following the cognitive interviews, a final meeting was conducted online with the SURP to ensure consensus on all changes to the questionnaire, including guidance for respondents on how to complete the questionnaire. Table 4 lists the 46 questionnaire items (including questions on socio-demographic and clinical background) used in the feasibility study.

**Table 4:** Classification of all 46 PREMAC items. Every item of the prototype questionnaire, showing its conceptual domain, response format, the population to which it applied, its intended measurement role, and whether it was entered into the exploratory factor analysis. Valid and non-applicable counts are over all 171 respondents.

Item	Actual Wording	Conceptual domain	Response format	Applicable population/ routing	Intended role	In EFA	Reason for inclusion/ exclusion	Valid n	NA n
	**First contact**								
Q1	How soon after developing a symptom did you seek help from a health service or individual?	Reasons for accessing AO	Ordered categorical (4 options)	All respondents	Categorical/descriptive item	No	Descriptive timing item; not a reflective experience item	169	2
Q2	If you did not seek help immediately, can you tell us why you delayed?	Reasons for accessing AO	Categorical + free text	Respondents reporting delay	Categorical/descriptive item	No	Nominal reasons; not a reflective experience item	144	27
Q3	Which service or individual did you first contact?	Patient preparedness	Categorical (single choice)	All respondents	Routing item	No	Routes to Section B (helpline) or Section C (place of care)	161	10
	**24-hour Acute Oncology Helpline**								
Q4	Before your cancer treatment, were you given the emergency phone number and an explanation?	Patient preparedness	Categorical (Yes/Partially/No)	Q3 = AO Helpline	Standalone service-experience indicator	No	3-level format, not the 4-point scale; limited applicability (n = 104)	104	67
Q5	Did you or a friend/relative ring the Helpline?	Patient preparedness	Categorical (single choice)	Q3 = AO Helpline	Categorical/descriptive item	No	Descriptive; not an experience rating	92	79
Q6	How long did you wait before speaking with a nurse or doctor?	Accessibility of care	Ordered categorical (5 options)	Q3 = AO Helpline	Standalone service-experience indicator	No	5-level timing scale, not the 4-point scale; limited applicability	105	66
Q7	Were you given enough help on the phone that resolved your problem?	Competence of care	4-point ordered (1 No, not at all – 4 Yes, definitely)	Q3 = AO Helpline	Standalone service-experience indicator	No	Applicable only to helpline callers (n = 93); would force listwise deletion of all non-helpline respondents	93	78
Q8	After talking to the helpline did you attend hospital?	Accessibility of care	Categorical (single choice)	Q3 = AO Helpline	Routing item	No	Routes to Section C (attended) or Section J (did not attend)	103	68
	**Place of care**								
Q9	Where did you go when you attended hospital?	Accessibility of care	Categorical (single choice)	Attended hospital	Categorical/descriptive item	No	Nominal setting variable	142	29
Q10	How long did you wait before a nurse or doctor examined you?	Accessibility of care	Ordered categorical (5 options)	Attended hospital	Standalone service-experience indicator	No	5-level timing scale, not the 4-point scale	138	33
Q11	Were you kept informed about how long you would have to wait?	Communication & decision-making	4-point ordered (1 No, not at all – 4 Yes, definitely)	Attended hospital	Candidate reflective subscale item/ standalone	No	The rationale for exclusion from the 18-item analysis was not documented; examined in sensitivity analysis (S5 Table, Panel C)	142	29
Q12	Were you/your family given enough time to explain your condition and concerns?	Communication & decision-making	4-point ordered (1 No, not at all – 4 Yes, definitely)	Attended hospital	Candidate reflective subscale item	No	The rationale for exclusion from the 18-item analysis was not documented; examined in sensitivity analysis (S5 Table, Panel C)	138	33
	**Care environment**								
Q13	Was the unit in which you received care clean?	Care environment	4-point ordered (1 No, not at all – 4 Yes, definitely)	Attended hospital	Candidate reflective subscale item	Yes	Care-environment content; 4-point scale; applicable to all attenders	144	27
Q14	Were you given enough privacy?	Care environment	4-point ordered (1 No, not at all – 4 Yes, definitely)	Attended hospital	Candidate reflective subscale item	Yes	Care-environment content	140	31
Q15	Did you feel safe in the place you were examined and treated?	Care environment	4-point ordered (1 No, not at all – 4 Yes, definitely)	Attended hospital	Candidate reflective subscale item	Yes	Care-environment content	144	27
	**Quality of information and care**								
Q16	Was your condition explained in a way you could understand?	Communication & decision-making	4-point ordered (1 No, not at all – 4 Yes, definitely)	Attended hospital	Candidate reflective subscale item	Yes	Information/communication content	143	28
Q17	Was the treatment you received explained in a way you could understand?	Communication & decision-making	4-point ordered (1 No, not at all – 4 Yes, definitely)	Attended hospital	Candidate reflective subscale item	Yes	Information/communication content	141	30
Q18	Were you given as much information as you would have liked?	Communication & decision-making	4-point ordered (1 No, not at all – 4 Yes, definitely)	Attended hospital	Candidate reflective subscale item	Yes	Information/communication content	143	28
Q19	Did staff involve you in decisions as much as you wanted?	Communication & decision-making	4-point ordered (1 No, not at all – 4 Yes, definitely) + 2 non-scaled options	Attended hospital	Candidate reflective subscale item	Yes	Shared decision-making content; non-scaled options treated as non-applicable	137	34
Q20	Did staff encourage you to ask questions?	Communication & decision-making	4-point ordered (1 No, not at all – 4 Yes, definitely)	Attended hospital	Candidate reflective subscale item	Yes	Information/communication content	144	27
Q21	Did you feel you were listened to?	Communication & decision-making	4-point ordered (1 No, not at all – 4 Yes, definitely)	Attended hospital	Candidate reflective subscale item	Yes	Information/communication content	143	28
Q22	Did you feel able to discuss emotions and worries?	Patient anxiety	4-point ordered (1 No, not at all – 4 Yes, definitely) + 1 non-scaled option	Attended hospital	Candidate reflective subscale item	Yes	Emotional support content; non-scaled option treated as non-applicable	132	39
Q23	Were you treated with respect?	Compassionate care	4-point ordered (1 No, not at all – 4 Yes, definitely)	Attended hospital	Candidate reflective subscale item	Yes	Compassion/dignity content	143	28
Q24	Did you have confidence in the nurses and doctors treating you?	Competence of care	4-point ordered (1 No, not at all – 4 Yes, definitely)	Attended hospital	Candidate reflective subscale item	Yes	Trust/confidence content	144	27
Q25	Did staff do everything possible to control your symptoms?	Competence of care	4-point ordered (1 No, not at all – 4 Yes, definitely)	Attended hospital	Candidate reflective subscale item	Yes	Perceived effectiveness of care	141	30
	**Carer’s role**								
Q26	Did a family member/friend accompany you?	Carer’s role	Categorical (single choice)	Attended hospital	Routing item	No	Routes to Section F (carer items) or Section G	141	30
Q27	Were your relative/friend given as much information as they needed?	Carer’s role	4-point ordered (1 No, not at all – 4 Yes, definitely)	Q26 = Yes	Carer-specific item	No	Applicable only to accompanied respondents (n = 93)	93	78
Q28	Was your relative/friend involved in decisions as much as you wanted?	Carer’s role	4-point ordered (1 No, not at all – 4 Yes, definitely)	Q26 = Yes	Carer-specific item	No	Applicable only to accompanied respondents (n = 91)	91	80
Q29	If your relative/friend was upset, did staff support and comfort them?	Carer’s role	4-point ordered (1 No, not at all – 4 Yes, definitely)	Q26 = Yes and carer upset	Carer-specific item	No	Doubly conditional; lowest applicability (n = 76)	76	95
	**At the end of the episode of care**								
Q30	Were you transferred to a ward or discharged home?	Service issues	Categorical (single choice)	Attended hospital	Routing item	No	Routes to Section H (ward) or Section I (discharge home)	141	30
	**Transfer to ward**								
Q31	Were you happy with the time taken to be transferred to a ward?	Service issues	4-point ordered (1 No, not at all – 4 Yes, definitely)	Q30 = Transferred to ward	Standalone service-experience indicator	No	Applicable only to admitted respondents (n = 45)	45	126
	**Discharge home**								
Q32	Were you happy with the time taken for you to be discharged home?	Service issues	4-point ordered (1 No, not at all – 4 Yes, definitely)	Section I (discharge home)	Standalone service-process indicator (provisional)	Yes	Included in EFA; loads with care-environment factor, not discharge factor – see Table 7	141	30
Q33	Were you given a clear explanation about treatment and care going forward?	Service issues	4-point ordered (1 No, not at all – 4 Yes, definitely)	Section I (discharge home)	Candidate reflective subscale item	Yes	Discharge-information content	144	27
Q34	Were you told what signs and symptoms to look out for?	Service issues	4-point ordered (1 No, not at all – 4 Yes, definitely)	Section I (discharge home)	Candidate reflective subscale item	Yes	Discharge-information content	140	31
Q35	Were you told who to contact if worried after going home?	Service issues	4-point ordered (1 No, not at all – 4 Yes, definitely)	Section I (discharge home)	Candidate reflective subscale item	Yes	Discharge-information content. NB raw data contained the misspelling ‘Yes, definitley’ (corrected in preparation)	143	28
Q36	Was the information printed or written down for you?	Service issues	4-point ordered (1 No, not at all – 4 Yes, definitely)	Section I (discharge home)	Candidate reflective subscale item	Yes	Discharge-information content	141	30
	**Your overall experience**								
Q37	How would you rate your overall experience of care?	Global rating	5-point verbal (Very poor – Very good) AS ADMINISTERED; paper version specifies a 1–10 numeric scale	All respondents	Global rating	No	Single global item; not part of a reflective subscale.	165	6
Q38	Please tell us more about problems or concerns experienced	Free text	Free text	All respondents	Free-text item	No	Qualitative	94	77
	**About you**								
Q39	Who completed this questionnaire?	Administrative	Categorical (single choice)	All respondents	Clinical or demographic item	No	Administrative	166	5
Q40	Which type of cancer do you have?	Clinical	Categorical (single choice)	All respondents	Clinical or demographic item	No	Clinical background	153	18
Q41	Other than cancer, do you have any other health conditions?	Clinical	Free text	All respondents	Clinical or demographic item	No	Clinical background	101	70
Q42	Is your gender identity the same as sex registered at birth?	Demographic	Categorical (single choice)	All respondents	Clinical or demographic item	No	Demographic	167	4
Q43	What is your gender?	Demographic	Categorical (single choice)	All respondents	Clinical or demographic item	No	Demographic	161	10
Q44	What is your religion?	Demographic	Categorical (single choice)	All respondents	Clinical or demographic item	No	Demographic	166	5
Q45	How would you describe yourself (sexual orientation)?	Demographic	Categorical (single choice)	All respondents	Clinical or demographic item	No	Demographic	162	9
Q46	What is your ethnic group?	Demographic	Categorical (single choice)	All respondents	Clinical or demographic item	No	Demographic	154	17

“NA n” combines responses that were blank and responses that were structurally non-applicable because of questionnaire routing. Items were entered into the exploratory factor analysis only where they used the four-point evaluative scale and were applicable to all respondents who attended hospital.

### 4.3 Stage 3: Feasibility study

The administration process using SMS texting and an online survey platform was found to be feasible. A total of 171 participants completed the PREMAC questionnaire over a 14-week period (7th May to 12th August 2024) out of 1258 eligible patients invited (response rate 14%). The majority identified as female (56.5%, n = 91 of 161 reporting gender). Of the 153 respondents who reported an ethnic group other than ‘prefer not to say’, 94.1% (n = 144) identified as White (English, Welsh, Scottish, Northern Irish or British; Irish; or another White background) and 5.9% (n = 9) identified as Asian, Black (African, Caribbean or British) or another ethnic group. The mean completion time was 14.5 minutes (SD = 18.86), with a median of 8 minutes and a range of 2.27–100 minutes. Most participants rated their overall care experience positively, with 82.4% selecting “Very good” and 9.7% selecting “Good.” Only 4.2% of participants rated their care as “Poor” or “Very poor.” Regarding waiting times, 65.2% selected the “I did not have to wait long” response, while a smaller proportion (2.9%) reported waiting over two hours before assessment. See Table 5 for responses to all question items.

**Table 5:** Frequency and percentage distribution of responses for selected experience questions across levels of agreement.

Item	Not at all		Not really		To some extent		Definitely		To some extent + Definitely	Total
	n	%	n	%	n	%	n	%	%	n
Q7	8	8.6	4	4.3	22	23.7	59	63.44	87.1	93
Q11	7	4.9	19	13.38	43	30.3	73	51.41	81.7	142
Q12	0	0	4	2.9	16	11.6	118	85.51	97.1	138
Q13	1	0.7	2	1.39	13	9.03	128	88.89	97.9	144
Q14	3	2.1	5	3.57	35	25	97	69.29	94.3	140
Q15	2	1.4	1	0.69	10	6.94	131	90.97	97.9	144
Q16	1	0.7	2	1.4	24	16.8	116	81.12	97.9	143
Q17	1	0.7	1	0.71	20	14.2	119	84.4	98.6	141
Q18	2	1.4	9	6.29	19	13.3	113	79.02	92.3	143
Q19	0	0	10	7.3	22	16.1	105	76.64	92.7	137
Q20	0	0	9	6.25	20	13.9	115	79.86	93.8	144
Q21	0	0	6	4.2	11	7.69	126	88.11	95.8	143
Q22	0	0	17	12.88	14	10.6	101	76.52	87.1	132
Q23	0	0	1	0.7	8	5.59	134	93.71	99.3	143
Q24	1	0.7	2	1.39	16	11.1	125	86.81	97.9	144
Q25	1	0.7	5	3.55	14	9.93	121	85.82	95.7	141
Q27	4	4.3	5	5.38	9	9.68	75	80.65	90.3	93
Q28	2	2.2	10	10.99	11	12.1	68	74.73	86.8	91
Q29	5	6.6	6	7.89	14	18.4	51	67.11	85.5	76
Q31	4	8.9	1	2.22	7	15.6	33	73.33	88.9	45
Q32	8	5.7	5	3.55	24	17	104	73.76	90.8	141
Q33	1	0.7	7	4.86	24	16.7	112	77.78	94.4	144
Q34	7	5	16	11.43	15	10.7	102	72.86	83.6	140
Q35	5	3.5	8	5.59	10	6.99	120	83.92	90.9	143
Q36	35	25	16	11.35	24	17	66	46.81	63.8	141

The data overall shows a high level of agreement and satisfaction across most of the experience questions.

To explore the dimensional structure of patient experience captured by the questionnaire, an EFA was conducted on the 18 evaluative items. Of the 171 respondents, 27 provided no data on any of these items: 22 had reported that they did not attend hospital following contact with the helpline and were routed past the relevant sections, so that these items were structurally non-applicable rather than missing, and five discontinued the questionnaire before reaching them. Of the 144 respondents who reached the relevant sections, a further 23 did not provide a scorable response to at least one item, giving a complete-case analysis sample of 121. Item-level valid, non-applicable and missing counts are reported in S5 Table. Responses were concentrated in the most positive category, with between 48.8% and 94.2% of respondents selecting the top category across the 18 items. Sampling adequacy was confirmed with a Kaiser-Meyer-Olkin (KMO) value of 0.876, and Bartlett’s test of sphericity was statistically significant (χ²(153) = 1519.82, p < 0.001), indicating the data were suitable for factor analysis (Table 6).

**Table 6:** Exploratory factor analysis sample and factorability.

Parameter	Value
Items entered into the exploratory factor analysis	18
Analysis sample (complete cases)	n = 121
Kaiser-Meyer-Olkin measure of sampling adequacy	0.876
Bartlett’s test of sphericity, χ² (df)	1519.82 (153)
Significance	p < 0.001

Sampling adequacy was assessed on the Pearson correlation matrix of the 18 evaluative items in the complete-case sample. Because the items are four-category ordinal variables with substantial ceiling effects, the factor analysis itself was based on a polychoric correlation matrix. A polychoric Kaiser-Meyer-Olkin value is not reported because it proved unstable after the smoothing required to make the polychoric matrix positive definite.

Parallel analysis based on polychoric correlations supported the retention of three common factors (Fig 1). The corresponding component-based analysis supported two components; the factor-based result is reported here because the study used common factor analysis rather than principal components analysis. The three-factor solution was readily interpretable, and the factors were moderately to strongly intercorrelated (r = 0.415 between Factors 1 and 2, r = 0.639 between Factors 1 and 3, and r = 0.336 between Factors 2 and 3), which supported the use of an oblique rotation. The first factor, information, communication, involvement and perceived care quality, comprised Q16–Q22 together with Q25 (symptom control). The second factor, care environment, dignity, trust and safety, comprised Q13–Q15 and Q23, together with Q24. The third factor, discharge information and preparedness, comprised Q33–Q36. Standardised pattern coefficients and communalities are presented in Table 7. The care environment items loaded strongly on the second factor, with pattern coefficients of 0.917 for Q13 (cleanliness), 0.833 for Q14 (privacy) and 0.818 for Q15 (feeling safe).

**Table 7:** Standardised pattern coefficients and communalities (h^2^) from the exploratory factor analysis of 18 PREMAC items (polychoric correlations, minimum residual extraction, oblimin rotation; n = 121).

Item	Item content	Factor 1	Factor 2	Factor 3	h²	Allocation
Q16	Explanation of condition	0.952			0.697	Factor 1
Q17	Explanation of treatment received	0.985			0.952	Factor 1
Q18	Information about condition and treatment	0.945			0.921	Factor 1
Q19	Involvement in decisions	0.912			0.853	Factor 1
Q20	Encouraged to ask questions	0.894			0.856	Factor 1
Q21	Feeling listened to by staff	0.760			0.935	Factor 1
Q22	Able to discuss emotions and worries	0.657			0.782	Factor 1
Q25	Symptom control	0.576			0.853	Factor 1
Q13	Cleanliness of the unit		0.917		0.762	Factor 2
Q14	Privacy		0.833		0.691	Factor 2
Q15	Feeling safe		0.818		0.729	Factor 2
Q23	Treated with respect		0.440		0.604	Factor 2
Q24	Confidence in staff	0.472	0.379	0.330	0.906	Factor 2 (provisional) ‡
Q33	Explanation about treatment and onward care			0.586	0.689	Factor 3
Q34	Advice about worsening symptoms			0.835	0.873	Factor 3
Q35	Advice about re-attending services			0.673	0.805	Factor 3
Q36	Printed information on discharge			0.713	0.349	Factor 3
Q32	Time taken to be discharged home	0.074	0.515	0.313	0.538	Standalone indicator §

Values are standardised pattern coefficients from an oblimin (oblique) rotation of a smoothed polychoric correlation matrix, extracted by minimum residual factoring; n = 121 complete cases. Items are ordered by provisional domain. Coefficients smaller than |0.30| are suppressed for readability, except for Q24 and Q32, for which all coefficients are shown; the full matrix is given in S6 Table. An item was regarded as loading on a factor where its pattern coefficient was ≥ 0.40, and as cross-loading where two or more coefficients were ≥ 0.30 and differed by < 0.20. ‡ Q24 cross-loaded across all three factors; its allocation to Factor 2 is provisional and based on item content, and is not statistically unambiguous. § Q32 loaded primarily on Factor 2 (0.515) with a secondary loading on Factor 3 (0.313); because its content concerns the timeliness of discharge rather than discharge information and preparedness, it is reported as a standalone service-process indicator. h² = communality. The factors were correlated (r = 0.415 for Factors 1 and 2; r = 0.639 for Factors 1 and 3; r = 0.336 for Factors 2 and 3), which supported the use of an oblique rotation. All allocations are provisional pending confirmatory factor analysis.

**Fig 1:**
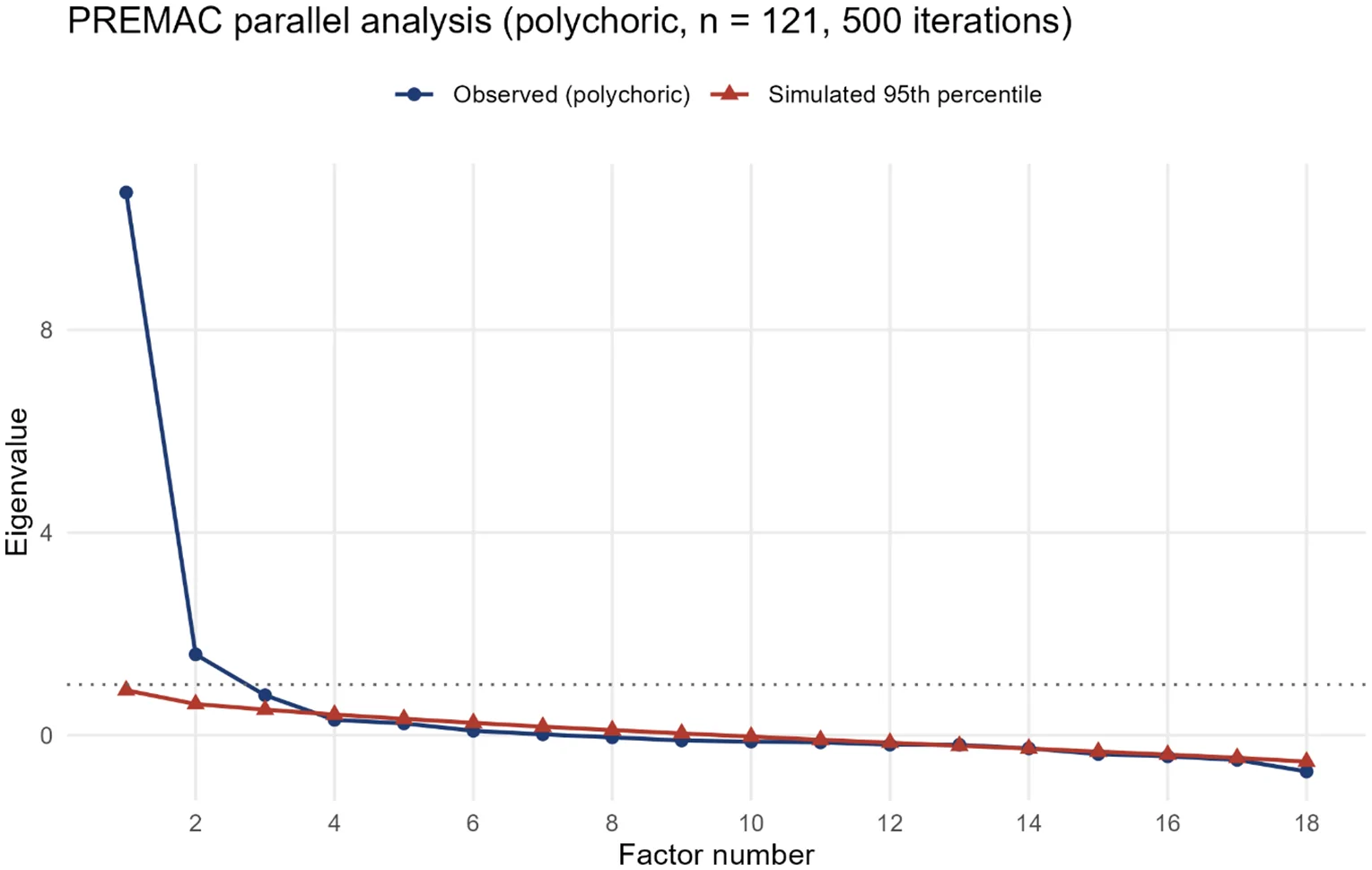
Parallel analysis for the PREMAC exploratory factor analysis.

Three items warranted additional consideration. Q24 (‘Confidence in staff’) cross-loaded across all three factors (0.472, 0.379 and 0.330). It was provisionally allocated to Factor 2 because its content concerns confidence and trust in clinicians, although this allocation was not statistically unambiguous. Q32 (‘Time taken to be discharged home’) loaded primarily on Factor 2 (0.515), with a secondary loading on Factor 3 (0.313). Because it assesses discharge timeliness rather than the discharge information and preparedness represented by Q33–Q36, it was retained as a standalone service-process indicator. Q36 (‘Printed information on discharge’) loaded 0.713 on Factor 3 but had a lower communality than the other discharge items ($h^{\text{2}}=\text{0}.\text{3}\text{4}\text{9}$). It was retained provisionally because written discharge information was considered important for the content coverage of this domain.

Internal consistency was α=0.935 for Factor 1, α=0.711 for Factor 2 and α=0.704 for Factor 3 (Table 8). Removing Q25 from Factor 1 reduced alpha to 0.929, while removing Q24 from Factor 2 reduced alpha to 0.660. Adding Q32 to Factor 3 resulted in only a small increase, from 0.704 to 0.712. The principal factor pattern was broadly similar across the sensitivity analyses (S4 and S5 Tables).

**Table 8:** Internal consistency of the proposed (provisional) PREMAC domains (n = 121).

Provisional domain	Items	Cronbach’s alpha	Sensitivity analysis
Factor 1: Information, communication, involvement and perceived care quality	Q16, Q17, Q18, Q19, Q20, Q21, Q22, Q25	0.935	α = 0.929 without Q25
Factor 2: Care environment, dignity, trust and safety	Q13, Q14, Q15, Q23, Q24	0.711	α = 0.660 without Q24; Q24 cross-loaded and is allocated provisionally
Factor 3: Discharge information and preparedness	Q33, Q34, Q35, Q36	0.704	α = 0.712 if Q32 is added
Standalone service-process indicator	Q32	–	Reported as a single item, not as part of a reflective domain

Raw Cronbach’s alpha, complete-case sample (n = 121). Alpha is reported as a reliability coefficient only and does not by itself establish structural validity. Ordinal alpha, McDonald’s omega, item-total statistics and alpha-if-item-deleted are reported in S6 Table.

## 5. Discussion

International guidance on cancer care emphasises the importance of patient experience [31], which has been associated with clinical effectiveness and patient safety [32]. A key purpose of PREMs is to measure patients’ experiences of care and thus drive quality improvement [33]. To our knowledge, PREMAC is the first study to develop a prototype PREM for use with patients who have accessed AO services.

This study undertook to complete the first development stages of PREMAC at one site, following the framework described by Pesudovs et al. [23]. We first developed a conceptual framework drawn from a scoping review [22] and qualitative interviews. Secondly, we collaborated with a service user group to co-design PREMAC, including its question items, item order, response options and instructions, and cognitively tested these before refining the instrument. Thirdly, we tested the validity, acceptability and feasibility of PREMAC with a sample of patients who had recently accessed AO services at a single site.

In addition to feasibility, preliminary psychometric testing was undertaken to assess the structure and reliability of the prototype questionnaire. Exploratory factor analysis identified three provisional domains: information, communication, involvement and perceived care quality; care environment, dignity, trust and safety; and discharge information and preparedness. These domains reflected key themes from the conceptual framework and showed preliminary evidence of internal consistency. The findings provide initial evidence of structural validity at a single site but do not establish that the instrument is ready for service comparison. The domains, item allocations and domain labels should therefore be regarded as provisional. A subsequent multisite PREMAC 2 study will further evaluate the instrument across healthcare organisations with different configurations of AO service delivery, including confirmatory factor analysis in an independent sample. Subject to successful validation, PREMAC could support local quality-improvement initiatives and longitudinal monitoring of patient experience. Its use for benchmarking between providers would require further validation, while use outside the United Kingdom would additionally require formal cross-cultural adaptation. The results of the factor analysis suggest existing question items may need to be re-ordered to align with the three factors identified, to improve the interpretability and coherence of the instrument. Q14 (‘*Were you given enough privacy?*’) loaded strongly on the care environment factor, and privacy is also a question item in other PREMs developed for ED [16,17,19] and the NCPES. The number of question items and consequent length of the questionnaire was a concern, because while longer PREMs tend to be more comprehensive [14], they can also reduce response rate [34]. Nevertheless, all items were felt to be required.

Within this study, eligible patients were identified electronically via a hospital-employed data analyst, and future studies would need to test the potential for this method of distribution to be replicated in other hospitals. A future economic evaluation of the recruitment model might also be appropriate. Overall, responses were very positive, with some variation between question items. Items with most positive responses were Q15 (‘*Did you feel safe in the place you were examined and treated?’)*, Q21 (‘*Did you feel you were listened to?*’) and Q24 (‘*Did you have confidence in the nurses and doctors treating you?’)*, with ≥96% reporting either ‘To some extent’ or ‘Definitely’. Items with fewest positive responses were Q7 (‘*Were you given enough help on the phone that resolved your problem?*’), Q31 (‘*Were you happy with the time taken to be transferred to a ward?*’) and Q36 (‘*Was the information printed or written down for you?*’), with between 9–25% of respondents reporting ‘Not at all’. These latter areas indicate areas of care on which to focus improvement, the effectiveness of which could be measured in subsequent uses of the PREM as well as variations with other service providers.

## 6. Limitations

There are several limitations to this study. First, all interview participants and SURP members (all female) identified as White British, and the feasibility sample was predominantly White. This limited ethnic diversity may have influenced both the conceptual framework and the psychometric findings. The study was also conducted at a single tertiary cancer centre in southern England, and the findings may not generalise to district general hospitals, other configurations of AO service delivery, or populations differing in socioeconomic position, first language or digital access. Multisite testing in more diverse samples, together with accessible formats, non-digital modes of administration, translation and cross-cultural evaluation, would be required before wider use.

Second, PREMAC was administered through an SMS link to an online questionnaire. This may have introduced selection bias because a small minority of otherwise eligible patients did not have a mobile telephone number recorded, while completion required digital access and confidence in using an online survey. Third, the response rate was 14% (171 of 1,258 invited). Respondents may therefore have differed systematically from non-respondents in characteristics relevant to patient experience, including satisfaction with care, illness severity, age, ethnicity, first language and socioeconomic position. The estimates may consequently not represent the full invited population. Because survey responses were anonymous and the research team held no identifiable data, respondents could not be compared with non-respondents using routinely collected characteristics. The anonymous design also prevented reminders from being sent, which may have contributed to the response rate. In addition, participants were required to review the study information and record consent before beginning the questionnaire, which may have increased the burden of participation. Response rates may be higher when PREMAC is administered routinely for service evaluation, and future studies should consider additional recruitment and administration methods.

Fourth, responses were generally positive and were concentrated in the most favourable category for most items. Such ceiling effects are common in patient-experience surveys but restrict variation, may reduce discrimination between services performing at a high level, and create sparse contingency tables that make polychoric correlation estimates less stable. Q36 (‘Printed information on discharge’) showed a more dispersed response distribution than the other items. The polychoric correlation matrix required smoothing before factor extraction, although sensitivity analyses produced a broadly similar factor pattern.

Fifth, the primary factor analysis was based on 121 complete cases, equivalent to approximately 6.7 respondents per item. This was a modest sample for estimating an 18-item, three-factor structure, particularly given the sparse response categories and strong ceiling effects. The three-factor solution, the provisional allocation of the cross-loading item Q24, and the treatment of Q32 as a standalone service-process indicator should therefore be evaluated in a larger independent sample. The subsequent PREMAC 2 study will compare the proposed and alternative measurement structures using confirmatory factor analysis.

Sixth, the 18 items included in the factor analysis primarily characterised the hospital-attendance episode, including discharge. Patients whose contact with the AO service was resolved by telephone were therefore not represented in the structural analysis. Items concerning the role of carers were also excluded because they applied only to respondents who attended with a carer and the number of applicable responses was insufficient for factor analysis. The possible development of a carer-related domain may warrant investigation in a subsequent study.

Finally, recruitment took place over 14 weeks. During this period, eligible patients may have accessed AO services more than once but were sent only one survey invitation. Although this reduced the burden associated with repeated invitations, the questionnaire therefore captured only one episode of care and may not represent variation in an individual’s experience across multiple contacts.

Only selected measurement properties were examined. The study provided preliminary evidence relating to content validity, comprehensibility, structural validity, internal consistency, feasibility and acceptability. It did not assess test–retest reliability, measurement error, responsiveness, construct validity through formal hypothesis testing, measurement invariance, cross-cultural validity or criterion validity. PREMAC should therefore be regarded as a prototype requiring further evaluation rather than as a fully validated instrument.

## 7. Conclusion

The PREMAC study contributes to the international focus on patient-centred care for people with cancer experiencing an oncological emergency. PREMAC appears to be the first patient-reported experience measure developed specifically for AO. Preliminary single-site testing suggests that the prototype is acceptable to patients, feasible to administer, and provides initial evidence of a provisional three-domain structure and internal consistency. Further investigation is required to confirm the proposed structure in a larger independent sample, assess reliability over time and responsiveness, develop and evaluate a scoring approach, and determine whether PREMAC can be implemented consistently across different AO service-delivery models and healthcare providers.

## Supporting information

S1 Table Content validity exercise: Mean responses. (DOCX)

S2 Table Comparison of the primary ordinal EFA and the ML–Promax sensitivity analysis. (DOCX)

S3 Table Missing-data and coding sensitivity analyses. (DOCX)

S4 Table Internal consistency sensitivity analyses, including ordinal alpha, McDonald’s omega, item-total statistics and alpha-if-item-deleted. (DOCX)

S5 Table Item-level valid, non-applicable and missing counts, and response distributions. (DOCX)

S6 Table Full pattern matrix, communalities and uniquenesses from the exploratory factor analysis. (DOCX)

S1 Script Annotated R analysis script. (DOCX)
