# Supplementary material for: Development and preliminary validation of a patient-report experience measure of acute oncology: The PREMAC study
Source: PLoS One. 2026 Oct 27;21(10):e0359781. doi: 10.1371/journal.pone.0359781 (PMC13712615; doi:10.1371/journal.pone.0359781)
Supplement: Supplementary file 1 [file pone.0359781.s001.docx]

**S1 Table: Content validity exercise: Mean responses**

| **No.** | **Question** | **Clarity**  **1 = Not Clear**  **2 = Somewhat clear**  **3 = Quite clear**  **4 = Highly clear** | **Relevance**  **1 = Not relevant**  **2 = Somewhat relevant**  **3 = Quite relevant**  **4 = Highly relevant** | **Importance**  **1 = Not important**  **2 = Somewhat important**  **3 = Quite important**  **4 = Highly important** | **Comments on questions and response options made by SURP members** |
| --- | --- | --- | --- | --- | --- |
| **Section A: When you first became worried about a symptom or problem related to your cancer or cancer treatment** | | | | | |
| 1 | How soon after developing a symptom or problem did you seek help from a health service or individual? | 4 | 3.25 | 3.25 | S1: If they waited a while before reaching out, are we interested in why they waited?  S2Not sure how this question is going to help format a PACC |
| 2 | Which service or individual did you first contact | 3.25 | 3.25 | 3.25 | S3: I’d add Acute Oncology 24 hour Helpline. |
| **Section B: If you called the acute oncology telephone helpline with symptoms that worried you** | | | | | |
| 3 | Before your cancer treatment started, were you given the emergency phone number and an explanation of how to contact the Acute Oncology team? | 4 | 3.25 | 3.25 |  |
| 4 | Did you ring the Helpline or did a friend or relative ring the Helpline for you? | 4 | 3.25 | 3.25 | S1: Should the question refer to the acute emergency number |
| 5 | After phoning the acute oncology emergency number, how long did you wait before speaking with a nurse or doctor? | 4 | 3.5 | 3.5 | S1: “you or your friend or relative”? |
| 6 | Were you given help and advice on the phone that resolved your problem? | 4 | 4 | 4 |  |
| **Section C: If you attended hospital to be seen either directly by the acute oncology team on a special cancer ward or assessment unit or by doctors and nurses in Accident & Emergency (could it say here…… “if a family member or friend / carer attended with you, you can give us feedback on their experience in Section K)** | | | | | |
| 7 | If you attended hospital to be examined by doctors and nurses for your symptom(s) or condition, where did you go? | 4 | 4 | 4 |  |
| 8 | After arriving at the hospital, how long did you wait before a nurse or doctor examined you? | 4 | 4 | 4 | S1: Is there an extra question around if a friend / family member was able to attend / wait with you? Could lead to a better feeling of patient reassurance / feeling safe |
| 9 | Were you given enough time to explain your condition and concerns? | 4 | 4 | 4 | S1: are we interested if they weren’t able to explain their condition eg. if so poorly that a relative / friend had to explain?  Is a comment box / tell us more required for “yes to some extent” or “no” to aid improvements?  S2: V V important |
| **Section D: Care environment** | | | | | |
| 10 | Was the unit in which you received care comfortable, clean and welcoming? | 4 | 4 | 4 | S1: Is a comment box / tell us more required for “yes to some extent” or “no” to aid improvements? |
| 11 | Were you given enough privacy when you were being examined and treated? | 4 | 4 | 4 | S1: Is a comment box / tell us more required for “yes to some extent” or “no” to aid improvements? |
| 12 | Did you feel safe? | 4 | 3.73 | 3.75 | S1: Is a comment box / tell us more required for “yes to some extent” or “no” to aid improvements?  S2: This has a huge impact on latent trauma after care has been given. I believe. |
| **Section E: Communication** | | | | | |
| 13 | Was your condition and treatment explained to you in a way you could understand? | 4 | 4 | 4 | S1: Is a comment box / tell us more required for “yes to some extent” or “no” to aid improvements?  S2: Maybe include if an option was given to tape the conversation. |
| 14 | Did you feel that you were listened to by the nurses and doctors? | 4 | 3.75 | 3.75 | S1: Is a comment box / tell us more required for “yes to some extent” or “no” to aid improvements?  S2: Very Important |
| **Section F: Compassion & respect** | | | | | |
| 15 | Were you able to discuss any worries and fears you had about your symptoms, condition or treatment? | 4 | 4 | 4 | S1: Is a comment box / tell us more required for “yes to some extent” or “no” to aid improvements?  S2: So so important to be allowed the space to address psychological concerns as so often ignored in a pushed for time environment |
| 16 | Were you treated with respect by the nurses and doctors? | 4 | 4 | 4 | S1: Is a comment box / tell us more required for “yes to some extent” or “no” to aid improvements?  S4: This question could be merged with 14 |
| **Section G: Information preferences** | | | | | |
| 17 | Were you given the right amount of information about your condition and treatment? | 3.25 | 4 | 4 | S1: Is a comment box / tell us more required if not enough? “What else would you have liked?”  S4 It is difficult for a patient to assess whether they were given the right amount of information as they wouldn’t know what information could have been given  S2: Also please mention the option to allow carers to leave when this is being given or invite them to listen as not all want to know or do and aren’t given that option if patient doesn’t want to know themselves…..Perhaps another question on this is relevant |
| **Section H: Shared decision-making** | | | | | |
| 18 | Did the nurses and doctors involve you in decisions about your care and treatment as much as you wanted to be? | 4 | 4 | 4 | S1: …involve you (and/or your family member / friend) ??  S2: And your relatives /carers given the opportunity to discuss these choices. |
| **Section I: Patient empowerment** | | | | | |
| 19 | Did the nurses and doctors encourage you to ask any questions you had? | 4 | 4 | 4 | S1: Is a comment box / tell us more required for “yes to some extent” or “no” to aid improvements? |
| 20 | Did you feel comfortable asking nurses and doctors questions about your care and treatment? | 4 | 3.25 | 3.25 | S1: Is a comment box / tell us more required for “yes to some extent” or “no” to aid improvements?  S2: Ask if psychological concerns are met. Give it a separate category.  S4: Question appears to repeat 15 |
| **Section J: Competence of care** | | | | | |
| 21 | Did you have confidence and trust in the nurses and doctors treating you? | 4 | 4 | 4 | S1: Is a comment box / tell us more required for “yes to some extent” or “no” to aid improvements?  S2: : This is a tough one as only too aware that there is rarely a choice and want the clinicians to like you! |
| 22 | Do you think the doctors and nurses did everything possible to control your symptoms? | 4 | 4 | 4 | S1: Definitely need a comment box / tell us more required for “yes to some extent” or “no” to aid improvements? What else could they have done? |
| **Section K: Carer’s role** | | | | | |
| 23 | Did a family member, friend or partner accompany you while you received care from the AO team? | 4 | 4 | 4 | S1: Is a comment box / tell us more required for “yes to some extent” or “no” to aid improvements?  S2: Where they invited to do so?? Another valid question perhaps. |
| 24 | Was your relative/ friend / partner involved with decisions about your care as much as you wanted them to be? | 4 | 3.75 | 3.75 | S1: Is a comment box / tell us more required for “yes to some extent” or “no” to aid improvements? |
| 25 | Did the nurses and doctors provide your relative / friend / partner with as much information about your condition and treatment as they needed? | 4 | 3.75 | 3.75 | S1: Is a comment box / tell us more required for “yes to some extent” or “no” to aid improvements? |
| 26 | If your relative / friend/ partner was upset, did a nurse or doctor support and comfort them? | 4 | 3.75 | 3.75 | S1: Is a comment box / tell us more required for “yes to some extent” or “no” to aid improvements? |
| **Section L: At the end of the episode of care** | | | | | |
| 27 | After being treated by the acute oncology team, were you transferred to an in-patient ward or discharged home? | 4 | 3.75 | 3.75 | S1: Is a comment box / tell us more required for “yes to some extent” or “no” to aid improvements? |
| **Section M: Transferred to an in-patient ward** | | | | | |
| 28 | Was your transfer to an in-patient ward managed in a timely way? | 4 | 4 | 4 | S1: Is a comment box / tell us more required for “yes to some extent” or “no” to aid improvements? |
| **Section N: Discharge home** | | | | | |
| 29 | Was your discharge home managed in a timely way? | 4 | 3.75 | 3.75 | S1: Is a comment box / tell us more required for “yes to some extent” or “no” to aid improvements? |
| 30 | Were you given a clear explanation about your treatment and care going forward and what you need to do to take care of yourself once at home? | 4 | 4 | 4 | S1: Is a comment box / tell us more required for “yes to some extent” or “no” to aid improvements? |
| 31 | Were you told what signs and symptoms to look out for after you had gone home? | 4 | 4 | 4 | S1: Is a comment box / tell us more required for “yes to some extent” or “no” to aid improvements? |
| 32 | Were you told who to contact if you were worried about your condition and treatment after you had gone home? | 4 | 4 | 4 | S1: Is a comment box / tell us more required for “yes to some extent” or “no” to aid improvements?  S2: Was all of the above written down? |
| **Section O: Your overall experience** | | | | | |
| 33 | How would you rate your overall experience of care | 3.5 | 3.75 | 3.75 | S1: Is this question about the time from reaching out or just about their time in AO Team |
| **Section P: About You** | | | | | |
| 34 | Who completed this questionnaire? | 4 | 3.75 | 3.75 |  |
| 35 | Which type of cancer do you have? | 4 | 4 | 4 |  |
| 36 | Other than cancer, do you have any physical or mental health condition? Please list | 4 | 3.75 | 3.75 | S4: I think there should be an “s” on the end of condition…s |
| 37 | Are you male or female? | 3.75 | 3.5 | 3.5 | S3: You may need to add a question eg “Are you the same sex that you were assigned at birth” |
| 38 | What is your religion? | 3.75 | 3.25 | 3.25 |  |
| 39 | Which of the following describes how you would describe yourself? | 3.75 | 3.25 | 3.25 |  |
| 40 | What is your ethnic group? | 3.75 | 2.75 | 2.75 |  |
| **Section P: Any other questions (free-text question)** | | | | | |
| 41 | Please add here anything else you would like to tell us about your experience with the AO team | 4 | 4 | 4 |  |
| **Please list any questions or topic areas that you think are missing from the list above.** | | | | | |
