# Supplementary material for: Development and preliminary validation of a patient-report experience measure of acute oncology: The PREMAC study
Source: PLoS One. 2026 Oct 27;21(10):e0359781. doi: 10.1371/journal.pone.0359781 (PMC13712615; doi:10.1371/journal.pone.0359781)
Supplement: Supplementary file 2 [file pone.0359781.s002.docx]

**S2 Table. Comparison of the primary ordinal EFA and the ML–Promax sensitivity analysis**

A sensitivity analysis using Pearson correlations, maximum-likelihood extraction and Promax rotation is presented alongside the primary ordinal EFA. Both used the same complete-case sample (n = 121) and both supported a three-factor solution.

| **Item** | **Item content** | **Ordinal F1** | **Ordinal F2** | **Ordinal F3** | **Ordinal h²** | **ML F1** | **ML F2** | **ML F3** | **ML h²** | **Comment** |
| --- | --- | --- | --- | --- | --- | --- | --- | --- | --- | --- |
| Q13 | Cleanliness of the unit | -0.10 | 0.92 | -0.03 | 0.76 | -0.08 | 0.49 | -0.10 | 0.15 | Same factor in both analyses; stronger coefficient in the ordinal EFA |
| Q14 | Privacy | 0.15 | 0.83 | -0.27 | 0.69 | 0.14 | 0.18 | -0.03 | 0.07 | Same factor in both analyses; markedly stronger coefficient in the ordinal EFA |
| Q15 | Feeling safe | -0.06 | 0.82 | 0.16 | 0.73 | -0.09 | 0.41 | 0.10 | 0.18 | Same factor in both analyses; stronger coefficient in the ordinal EFA |
| Q16 | Explanation of condition | 0.95 | -0.13 | -0.12 | 0.70 | 0.77 | -0.07 | -0.10 | 0.46 | Consistent |
| Q17 | Explanation of treatment received | 0.98 | -0.01 | -0.01 | 0.95 | 0.95 | -0.08 | -0.05 | 0.78 | Consistent |
| Q18 | Information about condition and treatment | 0.94 | -0.06 | 0.06 | 0.92 | 0.93 | 0.01 | -0.04 | 0.83 | Consistent |
| Q19 | Involvement in decisions | 0.91 | 0.04 | -0.01 | 0.85 | 0.84 | 0.01 | -0.03 | 0.69 | Consistent |
| Q20 | Encouraged to ask questions | 0.89 | 0.04 | 0.02 | 0.86 | 0.81 | -0.04 | 0.09 | 0.71 | Consistent |
| Q21 | Feeling listened to by staff | 0.76 | 0.06 | 0.25 | 0.94 | 0.74 | 0.12 | 0.10 | 0.78 | Consistent |
| Q22 | Able to discuss emotions and worries | 0.66 | 0.25 | 0.14 | 0.78 | 0.65 | -0.08 | 0.29 | 0.63 | Consistent |
| Q23 | Treated with respect | 0.28 | 0.44 | 0.25 | 0.60 | -0.02 | 0.48 | 0.13 | 0.31 | Same factor in both analyses |
| Q24 | Confidence in staff | 0.47 | 0.38 | 0.33 | 0.91 | 0.23 | 0.90 | -0.12 | 0.97 | Differs. ML–Promax places Q24 on F2 (0.902); the ordinal EFA shows a cross-loading across all three factors |
| Q25 | Symptom control | 0.58 | 0.27 | 0.27 | 0.85 | 0.42 | 0.67 | -0.12 | 0.83 | Differs. ML–Promax places Q25 on F2 (0.673); the ordinal EFA places it on F1 (0.576) |
| Q32 | Time taken to be discharged home | 0.07 | 0.52 | 0.31 | 0.54 | -0.03 | 0.44 | 0.10 | 0.24 | Consistent: both analyses place Q32 on F2 rather than the discharge factor |
| Q33 | Explanation about treatment and onward care | 0.25 | 0.13 | 0.59 | 0.69 | 0.19 | 0.31 | 0.32 | 0.50 | Differs. ML–Promax is ambiguous (0.312 / 0.318); the ordinal EFA is unambiguous (0.586) |
| Q34 | Advice about worsening symptoms | 0.19 | -0.09 | 0.83 | 0.87 | 0.10 | 0.07 | 0.71 | 0.66 | Consistent |
| Q35 | Advice about re-attending services | 0.25 | 0.10 | 0.67 | 0.81 | 0.11 | -0.24 | 0.99 | 0.86 | Same factor in both analyses. The very high ML–Promax pattern coefficient for Q35 was not reproduced in the ordinal EFA |
| Q36 | Printed information on discharge | -0.22 | -0.03 | 0.71 | 0.35 | -0.09 | 0.06 | 0.37 | 0.13 | Same factor in both analyses; stronger coefficient in the ordinal EFA, communality remains low |

*The ordinal EFA is the primary analysis because the items are four-category ordinal variables with substantial ceiling effects; the use of Pearson correlations for highly skewed ordinal items may attenuate estimated associations. Communalities are systematically lower in the ML–Promax columns, particularly for the care environment items.*
