# Supplementary material for: Development and preliminary validation of a patient-report experience measure of acute oncology: The PREMAC study
Source: PLoS One. 2026 Oct 27;21(10):e0359781. doi: 10.1371/journal.pone.0359781 (PMC13712615; doi:10.1371/journal.pone.0359781)
Supplement: Supplementary file 3 [file pone.0359781.s003.docx]

**S3 Table. Missing-data and coding sensitivity analyses**

Each sensitivity analysis used polychoric correlations, minimum residual extraction and oblimin rotation with three factors, as in the primary analysis. Panel C additionally includes Q11 and Q12.

**Panel A. Available-case (pairwise) polychoric estimation, n = 144**

| **Item** | **Item content** | **Factor 1** | **Factor 2** | **Factor 3** | **Communality** | **Primary** | **Cross-loading** |
| --- | --- | --- | --- | --- | --- | --- | --- |
| Q13 | Cleanliness of the unit | -0.080 | 0.904 | -0.015 | 0.743 | F2 | — |
| Q14 | Privacy | 0.107 | 0.861 | -0.228 | 0.718 | F2 | — |
| Q15 | Feeling safe | -0.037 | 0.810 | 0.146 | 0.728 | F2 | — |
| Q16 | Explanation of condition | 0.940 | -0.093 | -0.082 | 0.708 | F1 | — |
| Q17 | Explanation of treatment received | 0.937 | -0.020 | 0.039 | 0.912 | F1 | — |
| Q18 | Information about condition and treatment | 0.935 | -0.014 | 0.022 | 0.890 | F1 | — |
| Q19 | Involvement in decisions | 0.981 | 0.014 | -0.070 | 0.885 | F1 | — |
| Q20 | Encouraged to ask questions | 0.891 | -0.007 | 0.036 | 0.834 | F1 | — |
| Q21 | Feeling listened to by staff | 0.743 | 0.091 | 0.235 | 0.947 | F1 | — |
| Q22 | Able to discuss emotions and worries | 0.637 | 0.221 | 0.169 | 0.804 | F1 | — |
| Q23 | Treated with respect | 0.160 | 0.476 | 0.313 | 0.609 | F2 | Yes |
| Q24 | Confidence in staff | 0.451 | 0.413 | 0.239 | 0.846 | F1 | Yes |
| Q25 | Symptom control | 0.523 | 0.268 | 0.244 | 0.776 | F1 | — |
| Q32 | Time taken to be discharged home | 0.078 | 0.454 | 0.297 | 0.469 | F2 | — |
| Q33 | Explanation about treatment and onward care | 0.236 | 0.093 | 0.580 | 0.656 | F3 | — |
| Q34 | Advice about worsening symptoms | 0.138 | -0.076 | 0.872 | 0.894 | F3 | — |
| Q35 | Advice about re-attending services | 0.259 | 0.162 | 0.599 | 0.785 | F3 | — |
| Q36 | Printed information on discharge | -0.185 | -0.021 | 0.682 | 0.316 | F3 | — |

**Panel B. Non-scaled responses on Q19 and Q22 coded as the most positive category, n = 131**

| **Item** | **Item content** | **Factor 1** | **Factor 2** | **Factor 3** | **Communality** | **Primary** | **Cross-loading** |
| --- | --- | --- | --- | --- | --- | --- | --- |
| Q13 | Cleanliness of the unit | -0.150 | 0.921 | 0.030 | 0.762 | F2 | — |
| Q14 | Privacy | 0.165 | 0.847 | -0.289 | 0.737 | F2 | — |
| Q15 | Feeling safe | -0.004 | 0.806 | 0.090 | 0.700 | F2 | — |
| Q16 | Explanation of condition | 0.967 | -0.095 | -0.160 | 0.706 | F1 | — |
| Q17 | Explanation of treatment received | 0.973 | -0.048 | 0.012 | 0.923 | F1 | — |
| Q18 | Information about condition and treatment | 0.946 | -0.034 | 0.024 | 0.897 | F1 | — |
| Q19 | Involvement in decisions | 0.901 | 0.038 | 0.004 | 0.846 | F1 | — |
| Q20 | Encouraged to ask questions | 0.852 | 0.017 | 0.081 | 0.832 | F1 | — |
| Q21 | Feeling listened to by staff | 0.758 | 0.058 | 0.259 | 0.938 | F1 | — |
| Q22 | Able to discuss emotions and worries | 0.673 | 0.249 | 0.121 | 0.797 | F1 | — |
| Q23 | Treated with respect | 0.258 | 0.452 | 0.280 | 0.622 | F2 | — |
| Q24 | Confidence in staff | 0.482 | 0.397 | 0.296 | 0.898 | F1 | Yes |
| Q25 | Symptom control | 0.571 | 0.269 | 0.280 | 0.859 | F1 | — |
| Q32 | Time taken to be discharged home | 0.043 | 0.519 | 0.345 | 0.540 | F2 | Yes |
| Q33 | Explanation about treatment and onward care | 0.258 | 0.135 | 0.582 | 0.691 | F3 | — |
| Q34 | Advice about worsening symptoms | 0.161 | -0.084 | 0.865 | 0.897 | F3 | — |
| Q35 | Advice about re-attending services | 0.314 | 0.144 | 0.559 | 0.741 | F3 | — |
| Q36 | Printed information on discharge | -0.174 | -0.048 | 0.645 | 0.296 | F3 | — |

**Panel C. Analysis including Q11 and Q12, n = 118**

| **Item** | **Item content** | **Factor 1** | **Factor 2** | **Factor 3** | **Communality** | **Primary** | **Cross-loading** |
| --- | --- | --- | --- | --- | --- | --- | --- |
| Q13 | Cleanliness of the unit | -0.122 | 0.863 | 0.003 | 0.678 | F2 | — |
| Q14 | Privacy | 0.149 | 0.863 | -0.325 | 0.734 | F2 | — |
| Q15 | Feeling safe | -0.101 | 0.875 | 0.125 | 0.782 | F2 | — |
| Q16 | Explanation of condition | 0.875 | -0.080 | -0.029 | 0.693 | F1 | — |
| Q17 | Explanation of treatment received | 0.902 | 0.043 | 0.095 | 0.946 | F1 | — |
| Q18 | Information about condition and treatment | 0.862 | -0.046 | 0.204 | 0.931 | F1 | — |
| Q19 | Involvement in decisions | 0.824 | 0.081 | 0.092 | 0.831 | F1 | — |
| Q20 | Encouraged to ask questions | 0.802 | 0.075 | 0.139 | 0.839 | F1 | — |
| Q21 | Feeling listened to by staff | 0.734 | 0.091 | 0.300 | 0.936 | F1 | — |
| Q22 | Able to discuss emotions and worries | 0.603 | 0.271 | 0.213 | 0.786 | F1 | — |
| Q23 | Treated with respect | 0.182 | 0.445 | 0.349 | 0.590 | F2 | Yes |
| Q24 | Confidence in staff | 0.443 | 0.419 | 0.351 | 0.905 | F1 | Yes |
| Q25 | Symptom control | 0.500 | 0.322 | 0.339 | 0.847 | F1 | Yes |
| Q32 | Time taken to be discharged home | 0.029 | 0.557 | 0.331 | 0.570 | F2 | — |
| Q33 | Explanation about treatment and onward care | 0.273 | 0.169 | 0.559 | 0.676 | F3 | — |
| Q34 | Advice about worsening symptoms | 0.260 | -0.083 | 0.789 | 0.847 | F3 | — |
| Q35 | Advice about re-attending services | 0.283 | 0.108 | 0.659 | 0.792 | F3 | — |
| Q36 | Printed information on discharge | -0.147 | -0.024 | 0.624 | 0.310 | F3 | — |
| Q11 | Kept informed about waiting times | 0.452 | 0.469 | -0.040 | 0.563 | F2 | Yes |
| Q12 | Opportunity to explain condition and concerns | 1.011 | -0.104 | -0.292 | 0.750 | F1 | — |

*The principal factor pattern was stable across all three analyses: the information and communication items, the care environment items and the discharge items separated in the same way, and Q24 remained a cross-loading item in every analysis. In Panel C, Q12 aligned strongly with Factor 1 (1.011) and may be considered as a candidate Factor 1 item in PREMAC 2, whereas Q11 cross-loaded on Factors 1 and 2 (0.452 and 0.469) and is better treated as a standalone waiting-information indicator. Neither item was retrospectively added to the primary EFA model on the basis of the current data.*
