# Supplementary material for: Development and preliminary validation of a patient-report experience measure of acute oncology: The PREMAC study
Source: PLoS One. 2026 Oct 27;21(10):e0359781. doi: 10.1371/journal.pone.0359781 (PMC13712615; doi:10.1371/journal.pone.0359781)
Supplement: Supplementary file 4 [file pone.0359781.s004.docx]

**S4 Table. Internal consistency sensitivity analyses, including ordinal alpha, McDonald’s omega, item-total statistics and alpha-if-item-deleted**

**Panel A. Reliability of the provisional domains and sensitivity analyses (n = 121)**

| **Scale** | **Items** | **k** | **Raw α** | **Standardised α** | **Ordinal α** | **McDonald’s ω** |
| --- | --- | --- | --- | --- | --- | --- |
| Factor 1: Q16-Q22 + Q25 | Q16 Q17 Q18 Q19 Q20 Q21 Q22 Q25 | 8 | 0.935 | 0.940 | 0.977 | 0.978 |
| Factor 1: Q16-Q22 only | Q16 Q17 Q18 Q19 Q20 Q21 Q22 | 7 | 0.929 | 0.935 | 0.976 | 0.977 |
| Factor 1: Q16-Q22 + Q24 + Q25 | Q16 Q17 Q18 Q19 Q20 Q21 Q22 Q24 Q25 | 9 | 0.940 | 0.944 | 0.978 | 0.979 |
| Factor 2: Q13-Q15, Q23, Q24 | Q13 Q14 Q15 Q23 Q24 | 5 | 0.711 | 0.735 | 0.893 | 0.894 |
| Factor 2: without Q24 | Q13 Q14 Q15 Q23 | 4 | 0.660 | 0.675 | 0.873 | 0.877 |
| Factor 2: Q13-Q15 only | Q13 Q14 Q15 | 3 | 0.680 | 0.694 | 0.883 | 0.887 |
| Factor 2: + Q32 (sensitivity) | Q13 Q14 Q15 Q23 Q24 Q32 | 6 | 0.731 | 0.766 | 0.901 | 0.902 |
| Factor 2: + Q25 (alternative allocation) | Q13 Q14 Q15 Q23 Q24 Q25 | 6 | 0.762 | 0.783 | 0.908 | 0.909 |
| Factor 3: Q33-Q36 | Q33 Q34 Q35 Q36 | 4 | 0.704 | 0.772 | 0.871 | 0.884 |
| Factor 3: Q32-Q36 (alternative allocation) | Q32 Q33 Q34 Q35 Q36 | 5 | 0.712 | 0.764 | 0.863 | 0.875 |
| Factor 3: without Q36 | Q33 Q34 Q35 | 3 | 0.822 | 0.826 | 0.919 | 0.922 |

*Shaded rows are the provisional domains reported in Table 8 of the main manuscript. Raw and standardised alpha are based on Pearson covariances; ordinal alpha and omega are based on polychoric correlations and are therefore appreciably higher. None of these coefficients is evidence of structural validity. Removing Q36 raises the alpha of the discharge domain from 0.704 to 0.822; Q36 is nonetheless retained provisionally because written discharge information is conceptually important and identified the largest quality gap in the study.*

**Panel B. Item-total statistics and alpha if item deleted**

| **Domain items** | **Item** | **Item content** | **Corrected item-total r** | **α if item deleted** | **Scale α** |
| --- | --- | --- | --- | --- | --- |
| Q16 Q17 Q18 Q19 Q20 Q21 Q22 Q25 | Q16 | Explanation of condition | 0.620 | 0.937 | 0.935 |
| Q16 Q17 Q18 Q19 Q20 Q21 Q22 Q25 | Q17 | Explanation of treatment received | 0.833 | 0.926 | 0.935 |
| Q16 Q17 Q18 Q19 Q20 Q21 Q22 Q25 | Q18 | Information about condition and treatment | 0.871 | 0.919 | 0.935 |
| Q16 Q17 Q18 Q19 Q20 Q21 Q22 Q25 | Q19 | Involvement in decisions | 0.814 | 0.923 | 0.935 |
| Q16 Q17 Q18 Q19 Q20 Q21 Q22 Q25 | Q20 | Encouraged to ask questions | 0.819 | 0.923 | 0.935 |
| Q16 Q17 Q18 Q19 Q20 Q21 Q22 Q25 | Q21 | Feeling listened to by staff | 0.849 | 0.922 | 0.935 |
| Q16 Q17 Q18 Q19 Q20 Q21 Q22 Q25 | Q22 | Able to discuss emotions and worries | 0.746 | 0.931 | 0.935 |
| Q16 Q17 Q18 Q19 Q20 Q21 Q22 Q25 | Q25 | Symptom control | 0.740 | 0.929 | 0.935 |
| Q13 Q14 Q15 Q23 Q24 | Q13 | Cleanliness of the unit | 0.588 | 0.619 | 0.711 |
| Q13 Q14 Q15 Q23 Q24 | Q14 | Privacy | 0.476 | 0.688 | 0.711 |
| Q13 Q14 Q15 Q23 Q24 | Q15 | Feeling safe | 0.503 | 0.649 | 0.711 |
| Q13 Q14 Q15 Q23 Q24 | Q23 | Treated with respect | 0.414 | 0.697 | 0.711 |
| Q13 Q14 Q15 Q23 Q24 | Q24 | Confidence in staff | 0.476 | 0.660 | 0.711 |
| Q33 Q34 Q35 Q36 | Q33 | Explanation about treatment and onward care | 0.566 | 0.628 | 0.704 |
| Q33 Q34 Q35 Q36 | Q34 | Advice about worsening symptoms | 0.626 | 0.556 | 0.704 |
| Q33 Q34 Q35 Q36 | Q35 | Advice about re-attending services | 0.632 | 0.586 | 0.704 |
| Q33 Q34 Q35 Q36 | Q36 | Printed information on discharge | 0.349 | 0.822 | 0.704 |

*Q36 (shaded) is the only item whose removal would materially improve its domain. Q23 has the lowest corrected item-total correlation within the care environment domain (0.414), consistent with its position between two factors.*
