# Supplementary material for: Development and preliminary validation of a patient-report experience measure of acute oncology: The PREMAC study
Source: PLoS One. 2026 Oct 27;21(10):e0359781. doi: 10.1371/journal.pone.0359781 (PMC13712615; doi:10.1371/journal.pone.0359781)
Supplement: Supplementary file 5 [file pone.0359781.s005.docx]

**S5 Table. Item-level valid, non-applicable and missing counts, and response distributions**

Panel A covers all items using the four-point evaluative scale (n = 171 respondents). Panel B gives response distributions for the 18 items entered into the factor analysis, within the complete-case sample (n = 121).

**Panel A. Valid, non-scaled and missing responses**

| **Item** | **Item content** | **Valid** | **Non-scaled response** | **Blank** | **Not available** | **% valid** |
| --- | --- | --- | --- | --- | --- | --- |
| Q7 | Support given by helpline | 93 | 0 | 78 | 78 | 54.4 |
| Q11 | Kept informed about waiting times | 142 | 0 | 29 | 29 | 83.0 |
| Q12 | Opportunity to explain condition and concerns | 138 | 0 | 33 | 33 | 80.7 |
| Q13 | Cleanliness of the unit | 144 | 0 | 27 | 27 | 84.2 |
| Q14 | Privacy | 140 | 0 | 31 | 31 | 81.9 |
| Q15 | Feeling safe | 144 | 0 | 27 | 27 | 84.2 |
| Q16 | Explanation of condition | 143 | 0 | 28 | 28 | 83.6 |
| Q17 | Explanation of treatment received | 141 | 0 | 30 | 30 | 82.5 |
| Q18 | Information about condition and treatment | 143 | 0 | 28 | 28 | 83.6 |
| Q19 | Involvement in decisions | 137 | 6 | 28 | 34 | 80.1 |
| Q20 | Encouraged to ask questions | 144 | 0 | 27 | 27 | 84.2 |
| Q21 | Feeling listened to by staff | 143 | 0 | 28 | 28 | 83.6 |
| Q22 | Able to discuss emotions and worries | 132 | 10 | 29 | 39 | 77.2 |
| Q23 | Treated with respect | 143 | 0 | 28 | 28 | 83.6 |
| Q24 | Confidence in staff | 144 | 0 | 27 | 27 | 84.2 |
| Q25 | Symptom control | 141 | 0 | 30 | 30 | 82.5 |
| Q27 | Information given to carer | 93 | 0 | 78 | 78 | 54.4 |
| Q28 | Carer involved in decisions | 91 | 0 | 80 | 80 | 53.2 |
| Q29 | Support and comfort for carer | 76 | 0 | 95 | 95 | 44.4 |
| Q31 | Time taken to be transferred to a ward | 45 | 0 | 126 | 126 | 26.3 |
| Q32 | Time taken to be discharged home | 141 | 0 | 30 | 30 | 82.5 |
| Q33 | Explanation about treatment and onward care | 144 | 0 | 27 | 27 | 84.2 |
| Q34 | Advice about worsening symptoms | 140 | 0 | 31 | 31 | 81.9 |
| Q35 | Advice about re-attending services | 143 | 0 | 28 | 28 | 83.6 |
| Q36 | Printed information on discharge | 141 | 0 | 30 | 30 | 82.5 |

*“Non-scaled response” counts respondents who selected a response option that is not a point on the ordered scale: on Q19, “I did not want to be involved and was happy to take advice from doctors and nurses” or “I was not well enough to be involved in decisions about my care”; on Q22, “I did not have any worries”. These were treated as non-scorable in the primary analysis.*

**Panel B. Response distributions in the complete-case sample (n = 121)**

| **Item** | **Item content** | **Mean** | **SD** | **% top category** | **% bottom category** | **Skewness** | **Kurtosis** |
| --- | --- | --- | --- | --- | --- | --- | --- |
| Q13 | Cleanliness of the unit | 3.85 | 0.44 | 87.6 | 0.8 | -3.59 | 15.53 |
| Q14 | Privacy | 3.6 | 0.68 | 68.6 | 2.5 | -1.89 | 3.71 |
| Q15 | Feeling safe | 3.88 | 0.48 | 91.7 | 1.7 | -4.51 | 21.78 |
| Q16 | Explanation of condition | 3.81 | 0.41 | 81.8 | 0 | -1.9 | 2.56 |
| Q17 | Explanation of treatment received | 3.84 | 0.39 | 85.1 | 0 | -2.28 | 4.4 |
| Q18 | Information about condition and treatment | 3.72 | 0.62 | 79.3 | 1.7 | -2.41 | 5.74 |
| Q19 | Involvement in decisions | 3.69 | 0.61 | 76 | 0 | -1.74 | 1.78 |
| Q20 | Encouraged to ask questions | 3.73 | 0.58 | 79.3 | 0 | -1.97 | 2.69 |
| Q21 | Feeling listened to by staff | 3.84 | 0.47 | 88.4 | 0 | -2.97 | 7.95 |
| Q22 | Able to discuss emotions and worries | 3.66 | 0.68 | 77.7 | 0 | -1.71 | 1.32 |
| Q23 | Treated with respect | 3.93 | 0.28 | 94.2 | 0 | -4.5 | 21.61 |
| Q24 | Confidence in staff | 3.83 | 0.48 | 86 | 0.8 | -3.2 | 11.74 |
| Q25 | Symptom control | 3.79 | 0.55 | 85.1 | 0.8 | -2.82 | 7.89 |
| Q32 | Time taken to be discharged home | 3.58 | 0.82 | 73.6 | 5.8 | -2.02 | 3.19 |
| Q33 | Explanation about treatment and onward care | 3.72 | 0.59 | 78.5 | 0.8 | -2.18 | 4.45 |
| Q34 | Advice about worsening symptoms | 3.58 | 0.81 | 75.2 | 3.3 | -1.77 | 1.93 |
| Q35 | Advice about re-attending services | 3.76 | 0.65 | 85.1 | 2.5 | -2.86 | 7.73 |
| Q36 | Printed information on discharge | 2.94 | 1.21 | 48.8 | 21.5 | -0.61 | -1.26 |

*Ceiling effects exceed the conventional 15% criterion on every item. Q36 is the most evenly distributed item (48.8% in the top category, SD 1.21); the remaining 17 items are markedly negatively skewed.*
