# Supplementary material for: Development and preliminary validation of a patient-report experience measure of acute oncology: The PREMAC study
Source: PLoS One. 2026 Oct 27;21(10):e0359781. doi: 10.1371/journal.pone.0359781 (PMC13712615; doi:10.1371/journal.pone.0359781)
Supplement: Supplementary file 6 [file pone.0359781.s006.docx]

**S6 Table. Full pattern matrix, communalities and uniquenesses from the exploratory factor analysis**

Full, unsuppressed standardised pattern coefficients corresponding to Table 7 of the main manuscript. Polychoric correlations, minimum residual extraction, oblimin rotation; n = 121 complete cases.

| **Item** | **Item content** | **Factor 1** | **Factor 2** | **Factor 3** | **Communality** | **Uniqueness** | **Primary** | **Cross-loading** |
| --- | --- | --- | --- | --- | --- | --- | --- | --- |
| Q13 | Cleanliness of the unit | -0.100 | 0.917 | -0.026 | 0.762 | 0.238 | F2 | — |
| Q14 | Privacy | 0.150 | 0.833 | -0.267 | 0.691 | 0.309 | F2 | — |
| Q15 | Feeling safe | -0.061 | 0.818 | 0.156 | 0.729 | 0.271 | F2 | — |
| Q16 | Explanation of condition | 0.952 | -0.133 | -0.121 | 0.697 | 0.303 | F1 | — |
| Q17 | Explanation of treatment received | 0.985 | -0.015 | -0.006 | 0.952 | 0.048 | F1 | — |
| Q18 | Information about condition and treatment | 0.945 | -0.056 | 0.057 | 0.921 | 0.079 | F1 | — |
| Q19 | Involvement in decisions | 0.912 | 0.041 | -0.009 | 0.853 | 0.147 | F1 | — |
| Q20 | Encouraged to ask questions | 0.894 | 0.037 | 0.023 | 0.856 | 0.144 | F1 | — |
| Q21 | Feeling listened to by staff | 0.760 | 0.063 | 0.249 | 0.935 | 0.065 | F1 | — |
| Q22 | Able to discuss emotions and worries | 0.657 | 0.246 | 0.136 | 0.782 | 0.218 | F1 | — |
| Q23 | Treated with respect | 0.283 | 0.440 | 0.250 | 0.604 | 0.396 | F2 | — |
| Q24 | Confidence in staff | 0.472 | 0.379 | 0.330 | 0.906 | 0.094 | F1 | Yes |
| Q25 | Symptom control | 0.576 | 0.265 | 0.273 | 0.853 | 0.147 | F1 | — |
| Q32 | Time taken to be discharged home | 0.074 | 0.515 | 0.313 | 0.538 | 0.462 | F2 | — |
| Q33 | Explanation about treatment and onward care | 0.251 | 0.131 | 0.586 | 0.689 | 0.311 | F3 | — |
| Q34 | Advice about worsening symptoms | 0.188 | -0.093 | 0.835 | 0.873 | 0.127 | F3 | — |
| Q35 | Advice about re-attending services | 0.251 | 0.097 | 0.673 | 0.805 | 0.195 | F3 | — |
| Q36 | Printed information on discharge | -0.223 | -0.025 | 0.713 | 0.349 | 0.651 | F3 | — |

*“Primary” is the factor with the largest absolute pattern coefficient and is a purely statistical designation; it is not the same as the provisional allocation reported in Table 7. Q24 has its largest coefficient on Factor 1 but is allocated provisionally to Factor 2 on conceptual grounds, and Q32 has its largest coefficient on Factor 2 but is reported as a standalone service-process indicator. Factor correlations: r = 0.415 (Factors 1 and 2), 0.639 (Factors 1 and 3), 0.336 (Factors 2 and 3).*

**Variance accounted for**

|  | **Factor 1** | **Factor 2** | **Factor 3** |
| --- | --- | --- | --- |
| SS loadings | 7.122 | 3.408 | 3.265 |
| Proportion Var | 0.396 | 0.189 | 0.181 |
| Cumulative Var | 0.396 | 0.585 | 0.766 |
| Proportion Explained | 0.516 | 0.247 | 0.237 |
| Cumulative Proportion | 0.516 | 0.763 | 1.000 |

*Because the rotation is oblique the factors are correlated, so these sums of squared loadings overlap and the cumulative figure cannot be partitioned cleanly between factors. It should be read as an approximate indication only.*
