# Supplementary material for: Development and preliminary validation of a patient-report experience measure of acute oncology: The PREMAC study
Source: PLoS One. 2026 Oct 27;21(10):e0359781. doi: 10.1371/journal.pone.0359781 (PMC13712615; doi:10.1371/journal.pone.0359781)
Supplement: Supplementary file 7 [file pone.0359781.s007.docx]

**S1 Script. Annotated R analysis script**

The full analysis script, PREMAC_Pilot_Revised_Ordinal_EFA.R, is provided as a separate file. It reads the raw data, documents every coding decision, runs validation checks, produces the primary ordinal exploratory factor analysis and all sensitivity analyses reported here, generates every table programmatically, and records the R version, package versions and session information. It deliberately does not compute an overall PREMAC score, does not impute missing values, and does not reassign any item between factors.

**Software environment**

| **Component** | **Version** |
| --- | --- |
| R | 4.4.2 (2024-10-31 ucrt) |
| psych | 2.4.6.26 |
| GPArotation | 2024.3-1 |
| readxl | 1.4.3 |
| dplyr | 1.1.4 |
| ggplot2 | 4.0.0 |
| writexl | 1.5.1 |
| Random seed | 20250731 |
